# Disease Resistance Genetics and Genomics in Octoploid Strawberry

**DOI:** 10.1534/g3.119.400597

**Published:** 2019-08-16

**Authors:** Christopher R. Barbey, Seonghee Lee, Sujeet Verma, Kevin A. Bird, Alan E. Yocca, Patrick P. Edger, Steven J. Knapp, Vance M. Whitaker, Kevin M. Folta

**Affiliations:** * Horticultural Sciences Department, University of Florida, Gainesville FL,; † Graduate Program in Plant Molecular and Cellular Biology, University of Florida, Gainesville, FL,; ‡ Gulf Coast Research and Education Center, University of Florida, Wimauma, FL,; § Department of Horticulture,; ** Ecology, Evolutionary Biology and Behavior,; †† Department of Plant Biology, Michigan State University, East Lansing, MI, and; ‡‡ Department of Plant Sciences, University of California, Davis, CA

**Keywords:** Strawberry, Disease Resistance, R-gene, eQTL, Subgenome Dominance, RenSeq

## Abstract

Octoploid strawberry (*Fragaria ×ananassa*) is a valuable specialty crop, but profitable production and availability are threatened by many pathogens. Efforts to identify and introgress useful disease resistance genes (R-genes) in breeding programs are complicated by strawberry’s complex octoploid genome. Recently-developed resources in strawberry, including a complete octoploid reference genome and high-resolution octoploid genotyping, enable new analyses in strawberry disease resistance genetics. This study characterizes the complete R-gene collection in the genomes of commercial octoploid strawberry and two diploid ancestral relatives, and introduces several new technological and data resources for strawberry disease resistance research. These include octoploid R-gene transcription profiling, *d*N/*d*S analysis, expression quantitative trait loci (eQTL) analysis and RenSeq analysis in cultivars. Octoploid fruit eQTL were identified for 76 putative R-genes. R-genes from the ancestral diploids *Fragaria vesca* and *Fragaria iinumae* were compared, revealing differential inheritance and retention of various octoploid R-gene subtypes. The mode and magnitude of natural selection of individual *F. ×ananassa* R-genes was also determined via *d*N/*d*S analysis. R-gene sequencing using enriched libraries (RenSeq) has been used recently for R-gene discovery in many crops, however this technique somewhat relies upon *a priori* knowledge of desired sequences. An octoploid strawberry capture-probe panel, derived from the results of this study, is validated in a RenSeq experiment and is presented for community use. These results give unprecedented insight into crop disease resistance genetics, and represent an advance toward exploiting variation for strawberry cultivar improvement.

Cultivated strawberry (*Fragaria ×ananassa*) is an important specialty crop that is cultivated world-wide for its sweet and flavorful fruit. However, marketable yields and post-harvest quality are significantly affected by disease. The strawberry fruit presents a vulnerable target for microbial pathogens (Farzaneh *et al.* 2015), as it is soft, moist, carbohydrate rich, and subject to damage from forces as seemingly innocuous as rain (Herrington *et al.* 2011). Genetic disease resistance has been a long-standing breeding priority. While breeders have made progress in producing varieties with tolerance to some pathogens, growers remain dependent on exogenous crop protection strategies to reduce pathogen loads (Cordova *et al.* 2017).

Plant R-genes are mediators of resistance to specific pathogens via effector triggered immunity, which results in the hypersensitive response and cell death (Amil-Ruiz *et al.* 2011). R-genes require a high degree of regulation to maintain homeostatic transcript levels to mitigate off-target protein interactions (Hammond-Kosack and Jones 1997). For this reason, many classes of functional R-genes are expressed at low levels unless elicited by pathogens (Lai and Eulgem 2017), contributing to the challenges of R-gene genomic and functional annotation. About 60% of characterized plant R-genes contain nucleotide-binding (NB-ARC) and leucine-rich-repeat (LRR) domains, and are referred to NLR genes (Funk *et al.* 2018). Plant R-genes are frequent targets for genetic improvement via breeding and genetic engineering (Baumgartner *et al.* 2015; Djian-Caporalino *et al.* 2014), and gene editing methods may accelerate their introduction into already-elite varieties. However, progress has been hindered because relatively few R-genes conferring novel resistance have been characterized (Amil-Ruiz *et al.* 2011). This problem is appreciable in strawberry, where the genetic complexity of octoploid cultivars presents unique challenges for functional identification and cloning of causal variants. An analysis of diploid R-genes across the Rosaceae genus was previously conducted (Arya *et al.* 2014). New genetic resources for high-resolution genotyping in octoploid strawberry have resulted in the recent identification of several disease resistance loci (Mangandi *et al.* 2017; Nellist *et al.* 2019; Cockerton *et al.* 2018; Pincot *et al.* 2018; Salinas *et al.* 2018; Anciro *et al.* 2018; Roach *et al.* 2016; Verma *et al.* 2018). However, the specific genes mediating resistance in these QTL intervals typically remain unresolved, as genomic resources for octoploid strawberry have not kept pace with genetic mapping.

Cultivated strawberry shares common ancestors with the extant diploid species *F. vesca*, *F. iinumae*, *F. nipponica*, and *F. viridis* (Edger *et al.* 2019) . A high-quality octoploid strawberry genome has been recently developed (Edger *et al.* 2019), enabling new kinds analyses and improved resolution compared with previous studies involving *Fragaria* NLRs (Jia *et al.* 2015; Zhong *et al.* 2018). Analysis of this *F. ×ananassa* ‘Camarosa’ genome identified the repertoire of octoploid R-gene sequences and further demonstrated a general genomic retention bias toward *F. vesca*-like sequences (Edger *et al.* 2019).

This research compares R-genes from octoploid strawberry with its diploid ancestors and provides additional analysis into the genetic control of R-gene expression and retention patterns. Additional bias toward retention of *F. vesca*-like R-genes was detected in octoploid strawberry, beyond the bias observed in non-R-gene coding sequences. This finding provides insight into potential practical drivers of biased gene retention. Conserved domains were compared to describe specific R-gene phylogenic relationships. The octoploid genome was used to assemble 61 fruit transcriptomes, and used to discover subgenomic expression quantitative trait loci (eQTL) for R-genes expressed in octoploid fruit. Data from the octoploid ‘Camarosa’ strawberry gene expression atlas (Sánchez-Sevilla *et al.*, 2017) was also used to determine R-gene transcript accumulation throughout the strawberry plant.

Resistance gene enrichment and sequencing (RenSeq) is an advantageous method for sequencing R-genes (Andolfo *et al.* 2014), and is likely to be very useful for *de novo* resolution of causal mutations (Witek *et al.* 2016). This method can be used to identify casual mutations within existing disease resistance QTL. For this purpose, a novel octoploid strawberry RenSeq capture probe library was designed using the R-genes identified in this analysis. This panel was experimentally validated using the University of Florida breeding germplasm. The results demonstrate robust capture and resequencing of octoploid and diploid R-genes using only short second-generation sequence reads and with relatively deep genomic multiplexing.

This report characterizes the complete R-gene collection in the genomes of commercial octoploid strawberry and two diploid ancestral relatives, providing the genome-level resolution necessary for fully exploiting genetic disease resistance in strawberry. This research introduces several new technology and data resources that now may be applied in study of strawberry disease resistance.

## Materials and Methods

### Plant populations and genetic materials

Three pedigree-connected and segregating strawberry populations were created from crosses ‘Florida Elyana’ × ‘Mara de Bois’, ‘Florida Radiance’ × ‘Mara des Bois’, and ‘Strawberry Festival’ × ‘Winter Dawn’ (Figure S1). These cultivars and 54 progeny were selected for RNAseq and Istraw35 SNP genotyping analysis (Verma *et al.* 2017), and were used to identify expressed genes and R-gene eQTL. *De novo* assemblies of ‘Mara des Bois’ and ‘Florida Elyana’ were also used to help design RenSeq capture probes.

For RenSeq, 14 disease resistant octoploid cultivars and elite breeding lines were selected from the University of Florida breeding program, and supplemented with ‘Camarosa’ and with the ancestral diploid *F. vesca*. The RenSeq lines are *F. vesca* genotype Hawaii 4, ‘Camarosa’, Sweet Sensation ‘Florida127’, ‘Florida Elyana’, 11.28-34, 11.77-96, 11.98-41, 12.115-10, 12.121-5, 13.26-134, 13.42-5, 13.55-195, 14.100-58, 14.100-59, 14.101-154, and 14.101-225.

### Identification of R-genes in strawberry spp

R-genes were predicted from the strawberry octoploid ‘Camarosa’ draft genome “F_ana_Camarosa_6-28-17.rm” (Edger *et al.* 2019), the diploid *F. vesca* reassembly “Fragaria_vesca_v2.0.a2” (Tennessen *et al.* 2014), and the diploid *F. innumae* assembly “FII_r1.1” (Hirakawa *et al.* 2014). Domain-level analysis was performed using the CLC Genomics Workbench 11 HMM implementation to search for Pfam- v29 domains on translated gene models from all genomic and transcriptomic strawberry resources. Motif search was performed on all translated gene models, using 56 R-gene-associated motifs collected from (Van Ghelder and Esmenjaud 2016; Lukasik and Takken 2009; Jupe *et al.* 2012). The CLC Genomics Workbench 11 (CLC Bio, Denmark) pattern discovery tool was trained on a preliminary list of strawberry R-genes, and novel motifs were reiterated back to all protein models. The ncoils sequence analysis algorithm (Lupas *et al.* 1991) was used to detect coiled-coil domains, and the output was parsed into GFF3 format for protein list reannotation. BLAST2GO annotation (Conesa *et al.* 2005) was performed to assign putative functions to all genes and confirm sequence association with disease resistance in a cross-referenced database.

Protein models containing canonical R-gene domains (*e.g.*, NB-ARC domain) were selected for inclusion as R-genes, as were gene models with more common domains (*e.g.*, LRR) with supporting evidence of an R-gene-associated motif. BLAST2GO annotated disease resistance associated genes not meeting the domain and motif-level criteria were manually analyzed for potential inclusion, leading to the inclusion of many LRR-containing RLK putative R-genes.

### NB-ARC phylogenetic analysis

NB-ARC domains were extracted from *F. iinumae*, *F. vesca*, and *F*. ×*ananassa* ‘Camarosa’. The CIPRES Science Gateway (Miller *et al.* 2010) was utilized for full-length protein sequence alignment using MUSCLE v3.7 (Edgar 2004) and Maximum likelihood analysis using RAxML v8.2.10 (Stamatakis 2014). Tree construction was performed using the PROTGAMMA rate distribution model with 100 bootstrap replicates, and rooted with human APAF-1. This process was replicated five times using different random number seeds. Trees were visualized in CLC Genomic Workbench 11 with a 50% threshold bootstrap value. Word clouds were generated per clade based on the relative domain content of the full proteins.

### dN/dS analysis

*d*N and *d*S values were computed using a set of custom scripts (https://github.com/Aeyocca/ka_ks_pipe/). Orthologous genes between the *F. ×ananassa and F. vesca* v4 (Edger *et al.* 2017b) genomes were identified using the compara module in JCVI utilities library (Tang *et al.*, 2015). Filtering of the JCVI utilities output was performed using a custom Perl script to identify the best syntenic ortholog and best blast hit below e-value 1e-4. Alignment of each orthologous gene pair was performed using MUSCLE v3.8.31 (Edgar 2004), followed by PAL2NAL (v14) (Suyama *et al.* 2006) to convert the peptide alignment to a nucleotide alignment. Finally, *d*N and *d*S values were computed between those gene pairs using codeml from PAML Version 4.9h (Yang 2007) with parameters specified in the control file found in the GitHub repository listed above.

### Tissue-specific transcriptome analysis

Raw short read RNAseq libraries from various ‘Camarosa’ tissue (Sánchez-Sevilla *et al.* 2017) with the study reference PRJEB12420 were download from the European Nucleotide Archive (https://www.ebi.ac.uk/ena). The complete 54 library RNAseq experiment consisted of six independent green receptacle libraries, six white receptacle libraries, six turning receptacle libraries, six red receptacle libraries, three root libraries, three leaf libraries, and six achene libraries each for all corresponding fruit stages. Raw RNAseq reads were assembled to the ‘Camarosa’ reference using the same pipeline as previously described for fruit transcriptome population analysis. Expression values from biologically-replicated libraries were averaged. Clustvis (Metsalu and Vilo 2015) was used for tissue-based RNAseq clustering and heatmap visualization using correlation distance and average linkage with scaling applied using default parameters.

### Fruit transcriptome analysis

61 fruit transcriptomes were sequenced via Illumina paired-end RNAseq (Avg. 65million reads, 2x100bp), and consisted of parents and progeny from crosses of ‘Florida Elyana’ × ‘Mara de Bois’, ‘Florida Radiance’ × ‘Mara des Bois’, and ‘Strawberry Festival’ × ‘Winter Dawn’. Reads were trimmed and mapped to the *F*. ×*ananassa* octoploid ‘Camarosa’ annotated genome using CLC Genomic Workbench 11 (mismatch cost of 2, insertion cost of 3, deletion cost of 3, length fraction of 0.8, similarity fraction of 0.8, 1 maximum hit per read). Reads that mapped equally well to more than one locus were discarded from the analysis. RNAseq counts were calculated in Transcripts Per Million (TPM). Three-dimensional principle component analysis (PCA) was performed on all RNAseq assemblies, including two replicates of ‘Mara des Bois’ fruit harvested three years apart and sequenced independently (Figure S2). Transcript abundances were normalized via the Box-Cox transformation algorithm performed in R (R. Development Core Team 2014) prior to eQTL analysis. The BLAST2GO pipeline was used to annotate the full ‘Camarosa’ predicted gene complement.

### Genotyping and genetic association of octoploid fruit R-genes

The Affymetrix IStraw35 Axiom SNP array (Verma *et al.* 2017) was used to genotype 60 individuals, including six parental lines from three independent biparental RNAseq populations (Figure S1). Sequence variants belonging to the Poly High Resolution (PHR) and No Minor Homozygote (NMH) marker classes were included for association mapping. Mono High Resolution (MHR), Off-Target Variant (OTV), Call Rate Below Threshold (CRBT), and Other marker quality classes, were discarded and not used for mapping. Individual marker calls inconsistent with Mendelian inheritance from parental lines were removed. The *F. vesca* physical map was used to orient marker positions as current octoploid maps do not include a majority of the available IStraw35 markers. A genome-wide analysis study (GWAS) was performed using GAPIT v2 (Tang *et al.* 2016) performed in R. R-gene eQTL were evaluated for significance based on the presence of multiple co-locating markers of *p*-value < 0.05 after false discovery rate correction for multiple comparisons. *Cis* *vs.* *trans* eQTL determinations were made by corroborating known ‘Camarosa’ physical gene position with the eQTL position the *F. vesca* map. In the example case of *FaDRL28*, subgenomic localization was confirmed via BLAST of the associated markers to the correct ‘Camarosa’ homeologous chromosome.

### Subgenome dominance in octoploid strawberry R-genes

The closest homolog for each *F*. ×*ananassa* ‘Camarosa’ gene in either Fragaria_vesca_v2.0.a2.cds or FII_r1.1cds was determined via BLAST analysis (e-value threshold < 0.1, word size = 25, match = 1, mismatch = l, existence = 0, extension = 2). *F. vesca*-like and *F. iinumae*-like gene counts and TPMs were independently calculated for each octoploid chromosome. This process was performed first on all genes in the ‘Camarosa’ genome to establish the baseline gene retention and expression bias. This process was then repeated using only predicted NLR genes containing an NB-ARC domain, as

### RenSeq probe design and validation

A panel of 39,501 of 120mer-length capture probes were designed based on the set of discovered strawberry R-genes from *F. ×ananassa* ‘Camarosa’, *F. vesca* genotype Hawaii 4, *F. iinumae* genomes, and *de novo* fruit transcriptomes from *F. ×ananassa* ‘Mara des Bois’ and ‘Florida Elyana’. A proprietary algorithm was used to select for capture probes of ideal hybridization thermodynamics and screened for potential off-target capture in the intergenic regions of ‘Camarosa’ and *F. vesca* (Rapid Genomics LLC, Gainesville FL). Probes were designed to not span exon-exon junction, to facilitate cross-utility for both genomic and cDNA libraries (Figure S3). A minimum baseline of 1x probe coverage was provided across the length of every predicted R-gene coding sequence, and additional probes were designed against conserved R-gene domains in order to promote capture of unknown and divergent R-genes across diverse octoploid accessions. RenSeq capture was performed on genomic libraries from fifteen octoploid disease-resistant cultivars and advanced breeding selections, and *F. vesca*, based on conditions set by (Jupe *et al.* 2014), with optimizations provided by Rapid Genomics LLC. Captured libraries were sequenced via 16x multiplexed Illumina HiSeq (2 × 100bp) and mapped to their respective annotated genomic references using CLC Genomic Workbench 11 (CLCBio, Aarhus, Denmark) (Similarity fraction = 0.9, Length fraction = 0.9, Match score = 1, Mismatch cost = 2, Insertion cost = 3, Deletion cost = 3).

### Data availability

Supplementary figures, tables, files, and raw data are available at FigShare. Custom scripts used for performing *d*N/*d*S analysis are available at GitHub: https://github.com/Aeyocca/ka_ks_pipe/. Raw short read RNAseq data from fruit transcriptomes are available from the NCBI Short Read Archive under project SRP039356 (http://www.ncbi.nlm.nih.gov/sra/?term=SRP039356). Raw short read RNAseq data from the ‘Camarosa’ gene expression atlas (Sánchez-Sevilla *et al.*, 2017) are available at the European Nucleotide Archive (https://www.ebi.ac.uk/ena) with the study reference PRJEB12420. Results derived from these data are compiled in Table S1. File S1 contains a GFF3 file for annotating the octoploid genome (Edger *et al.* 2019) with R-genes and R-gene domains. Renseq probe sequences are provided in File S2. IStraw35 marker names, map positions, and genotype sequences used in eQTL analysis are available in File S3. Supplemental material available at FigShare: https://doi.org/10.25387/g3.8143961.

## Results

### Octoploid and diploid R-genes

The genomes of octoploid ‘Camarosa’, diploid *F. vesca*, and diploid *F. iinumae* were analyzed for R-gene signatures. The *F.iinumae* genome was selected to represent the closely-related ‘old world’ diploid ancestors *F.iinumae*, *F. nipponica* and *F. viridis*, which each have highly similar but fragmented genomic assemblies.

Putative R-genes were identified based on protein domain and motif analysis, which identified gene models with traditional NLR-type domains, including coiled coil (CC), Toll Interleukin Receptor-like (TIR), Leucine Rich Repeat (LRR), and Nucleotide Binding - APAF-1 (apoptotic protease-activating factor-1), R proteins and CED-4 (*Caenorhabditis elegans* death-4 protein) (NBS, or NB-ARC). Gene models with NLR-type domains that are not highly specific to NLR sequences (*e.g.*, LRR domains) were included if there was also supporting evidence of an additional NLR-associated motif. BLAST2GO annotated disease resistance associated genes not meeting these criteria were analyzed manually, leading to the intentional inclusion of many putative Receptor-like Kinase (RLK-type) R-genes in this analysis.

**Figure 1:**
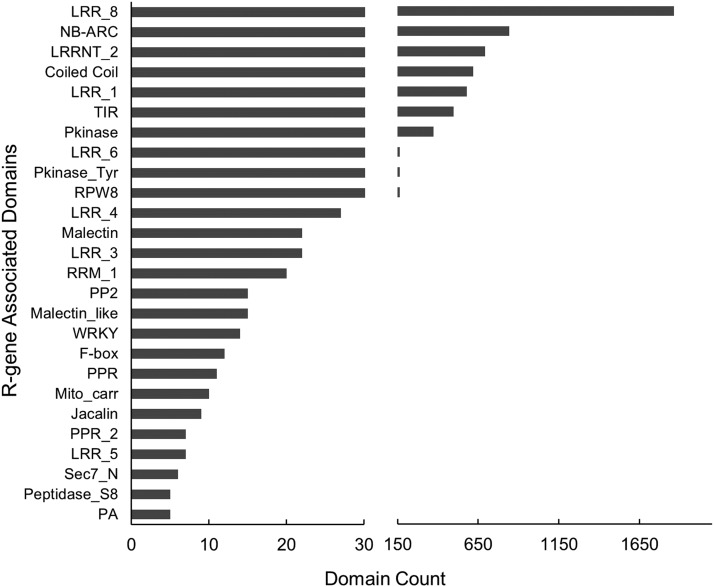
Canonical and non-Canonical R-Gene Domains in octoploid ‘Camarosa’. Classic TNL/CNL-type R-gene domains (TIR, NB-ARC, LRR, etc.) comprise the majority of domain classes in predicted R-genes, however a number of atypical domains are observed in high frequency. Domains below a count of five are not shown.

Octoploid *F*. ×*ananassa* ‘Camarosa’ carries 1,962 putative resistance genes (1.82% of all genes) (Table S1), including 975 complete or truncated NLR genes (Table 1). NLR gene content is similar in genic proportion to the 367 complete or truncated NLR genes in *F. vesca* (1.09% of all genes) and 387 in *F. iinumae* (0.5% of all genes). Traditional NLR domains comprise the majority of domain classes in all predicted resistance gene models in diploid and octoploid strawberry accessions (Figure 1). In many categories, the three genomes show somewhat dissimilar ratios of relative NLR-subtype content (Table 1). These include biases toward TIR-only proteins in *F. vesca* and CNL-type (CC-containing) NLR genes in *F. innumae*. Octoploid ‘Camarosa’ is proportionally intermediate for many NLR categories relative to *F. vesca and F. innumae*. A high proportion of TIR-NBS and TIR-NBS-LRR-containing genes is observed in ‘Camarosa’. However, the overall proportion of TNL-type (TIR-containing) NLR genes is similar when including TIR-only truncations. The Resistance to Powdery Mildew 8 (RPW8) domain, a disease resistance domain associated with broad-spectrum mildew resistance in Arabidopsis, appears frequently in strawberry and is present in 136 (13.9%) of octoploid NLRs (Table 1). Basic trends in NLR-subtype genomic content in ‘Camarosa’ does not more strongly resemble either *F. vesca* or *F. innumae*.

**Table 1:** NLR-gene subtype distribution across three strawberry species

	*F × ananassa* ‘Camarosa’	*F. vesca*	*F. iinumae*
NBS	193 *(19.8%)*	65 *(17.7%)*	45 *(11.6%)*
NBS-LRR	22 *(2.3%)*	7 *(1.9%)*	20 *(5.2%)*
**NBS-only type**	**215 *(22.1%)***	**72 *(19.6%)***	**65 *(16.8%)***
CC-NBS	163 *(16.6%)*	45 *(12.3%)*	126 *(32.6%*)
CC-NBS-LRR	30 *(3.1%)*	9 *(2.5%)*	8 *(2.1%)*
**CNL-type**	**192 *(19.7%)***	**54 *(14.7%)***	**134 *(34.6%)***
RPW8	91 *(9.3%)*	40 *(10.9%)*	23 *(5.9%)*
RPW8-NBS	45 *(4.6%)*	31 *(8.4%)*	13 *(3.4%)*
**RPW8-type**	**136 *(13.9%)***	**71 *(19.3%)***	**36 *(9.3%)***
TIR	134 *(13.7%)*	136 *(37.1%)*	86 *(22.2%)*
TIR-NBS	195 *(20.0%)*	18 *(4.9%)*	51 *(13.2%)*
TIR-NBS-LRR	103 (10.6%)	16 *(4.4%)*	15 *(3.9%)*
**TNL-type**	**432 *(40.3%)***	**170 *(46.3%)***	**152 *(39.3%)***
Total	975	367	387

Absolute and relative distribution of NLR-gene subtypes and truncated subtypes are shown. Domain combinations are shown for coiled coil (CC), toll interleukin receptor-like (TIR), leucine rich repeat (LRR), and nucleotide binding - APAF-1 (apoptotic protease-activating factor-1), R proteins and CED-4 (*Caenorhabditis elegans* death-4 protein) (NBS, or NB-ARC), and resistance to powdery mildew 8 (RPW8) domains.

In the ‘Camarosa’ genome, 750 of 975 NLR genes contain at least one NB-ARC domain, which is the most characteristic domain of NLR-type R-genes (Table 1). The ratio of ‘Camarosa’ NB-ARC-containing genes to total predicted gene content (1:144) is higher than in *F. vesca* (1:171) and *F. iinumae* (1:262), possibly indicating diversifying selection of NLR genes in octoploid *F*. ×*ananassa*. A substantial number of atypical domains are present on strawberry R-genes, including malectin-like carbohydrate-binding domains, RNA-binding domains, transcription factor-like WRKY and F-box domains, and several types of protein kinase domains (Figure 1).

Tandem clusters of R-genes were observed in all three of the analyzed strawberry genomes. The phenomena of R-gene expansion through tandem duplication is exemplified in the RPW8-containing R-gene class. Of the seventy-one RPW8-containing R-genes in *F. vesca*, all but seven reside in one of a few genomic clusters (Figure S4A-B). The major RPW8 cluster observed in *F. vesca* chromosome 1 is strongly retained in ‘Camarosa’ (Figure S4C). Similar R-gene hotspots are observed throughout the diploid and octoploid strawberry reference genomes. Genome annotations for all R-genes and NLR domains in ‘Camarosa’ are provided in File S1. Annotations are also available on the JBrowse web-based genome browser at the Genome Database for the Rosaceae (www.rosaceae.org).

### Phylogenetic analysis of strawberry NLRs

**Figure 2:**
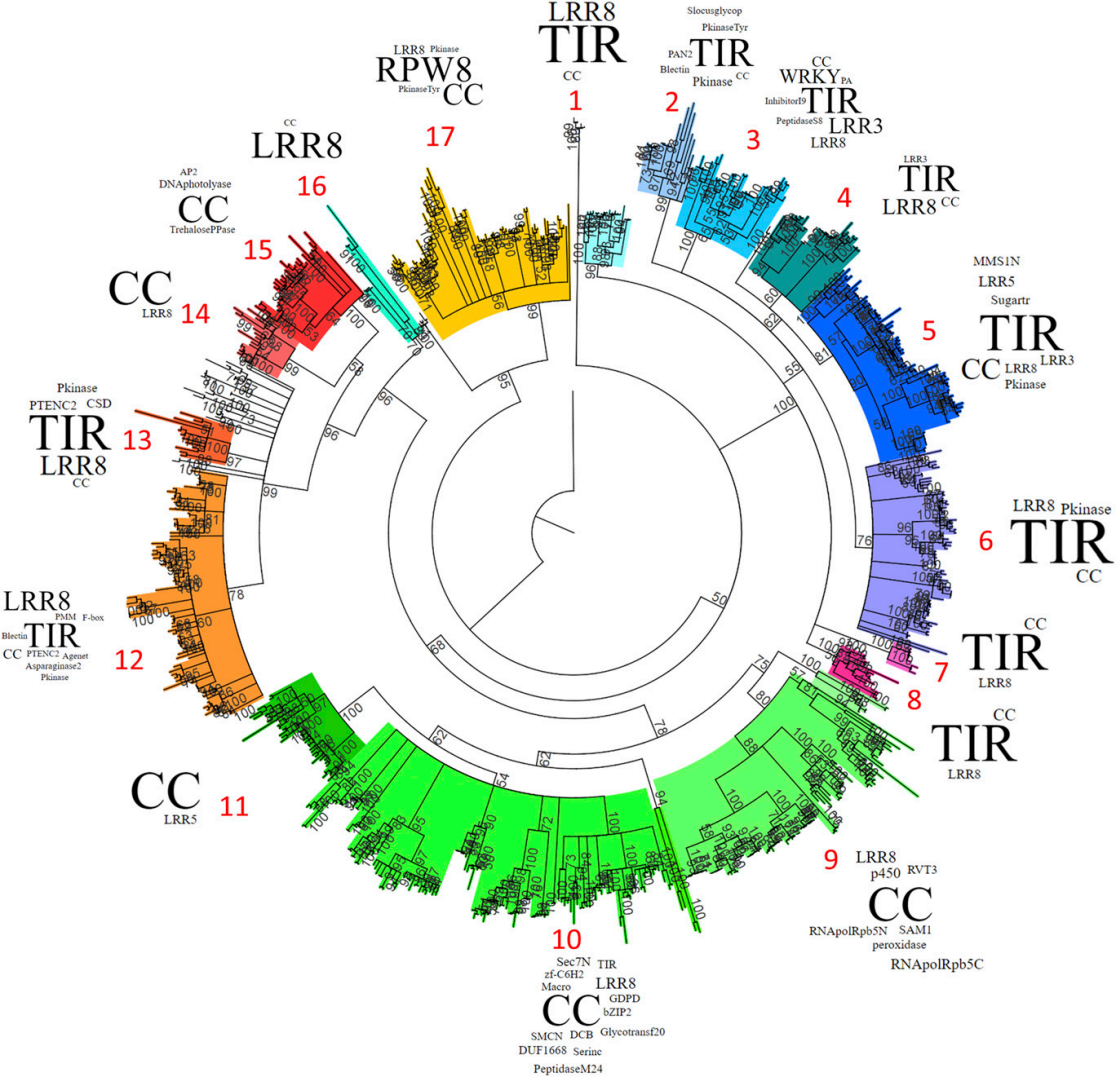
Phylogenetic Relationship of NB-ARC domains in *F. vesca*, *F. iinumae*, & *F. × ananassa* ‘Camarosa’. A. Full-length NB-ARC domains from strawberry spp. organize into clades based on NLR-gene subtype (CC, TIR, NB-ARC, LRR, and RPW8-containing combinations). Maximum likelihood bootstrap values (100 replicates) above a threshold of 50% are shown with the NB-ARC domain from human *Apaf1* as the outgroup. Clades are delineated by color and number (red). Word sizes correspond to relative domain content within each clade.

The conserved NB-ARC domains from ‘Camarosa’, *F. vesca*, and *F. iinumae* were compared via maximum likelihood analysis to examine evolutionary trends among NLR genes. NB-ARCs from all three genomes phylogenetically organized mostly according to their extended R-gene domain structures, with TNLs, CNLs, and RPW8-associated NB-ARCs forming clades based on this criteria (Figure 2). Minor NLR subtypes, such as WRKY-associated NLR genes, also sorted into a unique subclade based only on NB-ARC sequence. Multiple distinct clades with identical domain architectures were detected, and in a few cases these subclades are relatively distant from one another.

### R-gene transcript accumulation

**Figure 3:**
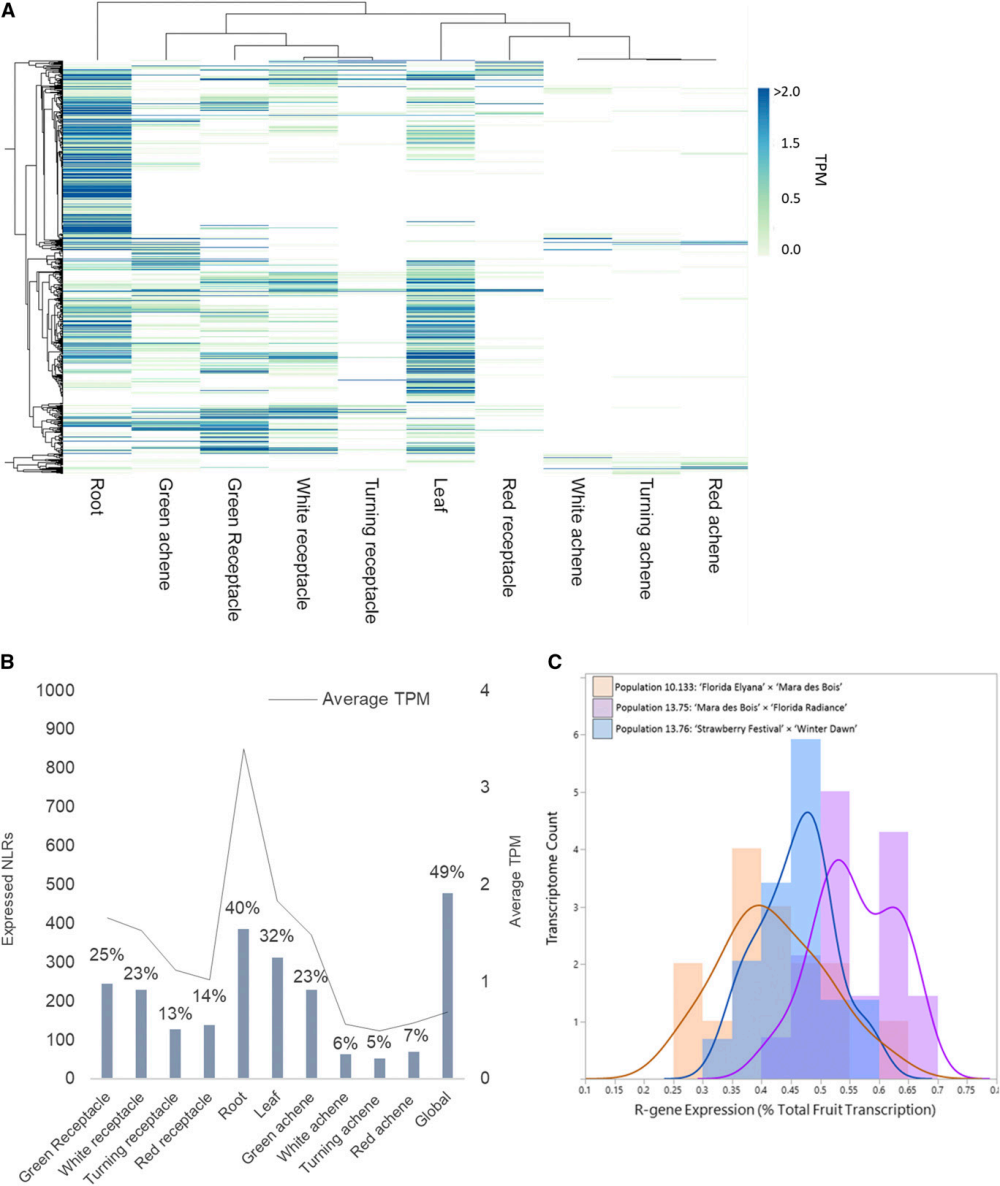
RNAseq-based Detection of Octoploid NLR Transcripts. A. Tissue-based heatmap of transcript accumulation (TPM) of NLRs in ‘Camarosa’. B. Total and proportional number of expressed NLRs (TPM >1) (left y-axis) with averaged ‘Camarosa’ transcript abundance (right y-axis), per tissue and in all tissues (global). C. Generalized R-gene mature-fruit expression across three segregating populations (n = 61).

Raw RNAseq expression data from different tissues of ‘Camarosa’, derived from the octoploid strawberry gene expression atlas (Sánchez-Sevilla *et al.* 2017), were reassembled based on the homeolog-level octoploid ‘Camarosa’ genome assembly (Edger *et al.* 2019). Out of 975 ‘Camarosa’ genome-predicted NLRs, 478 NLRs show evidence of RNAseq expression in any ‘Camarosa’ tissue (>1 Transcript per Million, TPM). A majority of ‘Camarosa’ NLR genes are predominantly expressed in the roots and leaves (Figure 3A-B). Comparatively few NLRs are predominantly or specifically expressed in the mature receptacle (139 expressed NLRs). Many NLR type R-genes are broadly specific to only one or two tissues. Expressed NLR genes from root, leaf, green and white receptacles show fairly poor overlap. Overall NLR transcript accumulation is correlated with ripening, with strongest expression in the earlier stages and decreasing with maturity in both the receptacle and achene. Complete R-gene expression values for each tissue are provided in Table S1.

Mature receptacle transcriptomes from 61 field-grown individuals of three octoploid populations reveal broadly stable R-gene expression levels (SD 0.09). R-genes comprise 1.8% of the predicted gene models in the ‘Camarosa’ genome, but represent an average of only 0.48% of total transcripts in the mature receptacle (Figure 3C). Minute but statistically significant absolute differences were observed between each of the three populations [F (2, 59) = 19.06, *P* < 0.00001]. To explore possible biases in the gene expression analysis caused by confounding environmental factors, principal component analysis (PCA) was performed on all RNAseq assemblies including two replicates of ‘Mara des Bois’ fruit harvested in different seasons (Figure S2). Total transcript-accumulation variation clusters most strongly according to familial relationship, with the ‘Mara des Bois’ replicates showing similar expression patterns. A measured amount of variation due to environmental influence can also be seen, as the two ‘Mara des Bois’ RNAseq replicates cluster somewhat more closely with their co-harvested progeny.

### R-gene eQTL in 61 strawberry fruit transcriptomes

eQTL analysis was performed to evaluate heritable genotypic effects on R-gene transcription, using 61 mature fruit octoploid transcriptomes and octoploid genotypes from the IStraw35 SNP array (Verma *et al.* 2017). This analysis identified 76 R-gene-like sequences with at least one highly-significant locus explaining differential expression (3.9% of octoploid genome-predicted R-genes). These R-genes include 39 NB-ARC containing genes, comprised of 16 TNL’s, 13 CNL’s, 3 NBS-RPW8 proteins, and 5 NB-ARC-only proteins, and an additional 6 RPW8-only genes and 4 TIR-only R-genes (Table S1). The majority of the remaining eQTL genes are LRR-containing RLK putative R-genes. As the ‘Camarosa’ genomic locus of each transcript is known, *cis* *vs.* *trans* eQTL status was determined. Of 76 significant R-gene eQTL transcripts, 52 R-genes are regulated via a *cis*-genetic locus (Table 2), and 24 R-genes are under regulation of both *cis* and *trans*-eQTL (Table 3). No solely *trans*-eQTL were discovered among this set of R-genes. The most significant IStraw35 SNP marker name and position for each R-gene transcript is provided with the eQTL phase, minor allele frequency, *p*-value (FDR-adjusted), heritability estimate, expression in parental lines, and BLAST2GO description.

**Table 2:** *Cis* eQTL pertaining to fruit-expressed genes in *F. ×ananassa*

R-gene Name	eQTL phase	IStraw35 AX-	MAF	*p*-value (FDR)	h^2^ estimate	Mara	Elya-na	Radi-ance	Festi-val	Winter dawn	Description
augustus_masked-fvb1-2-processed-gene-27.2	cis	89817565	0.43	0.0418	30.4%	36.6	113.3	32.2	21.3	34.3	uncharacterized protein LOC101293711
augustus_masked-fvb1-4-processed-gene-7.11	cis	166502627	0.16	0.0150	43.4%	2.3	0.6	4.0	3.8	1.6	PLT5_ARATH Sugar-proton symporter PLT5
augustus_masked-fvb2-4-processed-gene-105.5	cis	166503861	0.29	0.0114	80.9%	0.0	0.0	2.8	1.8	1.3	TMVRN_NICGU TMV resistance N
augustus_masked-fvb3-1-processed-gene-107.3	cis	166511589	0.50	0.0294	41.8%	0.0	0.5	0.7	0.8	0.3	DRL30_ARATH Probable disease resistance At5g04720
augustus_masked-fvb3-3-processed-gene-283.7	cis	89786873	0.38	0.0009	30.4%	0.8	0.4	0.0	2.6	0.4	DRL1_ARATH Probable disease resistance At1g12280
augustus_masked-fvb3-3-processed-gene-38.8	cis	166513199	0.23	0.0032	81.7%	6.0	3.0	5.5	0.8	4.9	Y4294_ARATH LRR receptor-like serine threonine- kinase
augustus_masked-fvb3-4-processed-gene-19.4	cis	166504873	0.28	0.0014	100%	0.1	4.8	5.6	8.0	0.1	DR100_ARATH DNA damage-repair toleration DRT100
augustus_masked-fvb4-1-processed-gene-166.2	cis	166505902	0.43	0.0083	91.5%	0.6	0.4	2.5	1.4	1.8	MKKA_DICDI Mitogen-activated kinase kinase
augustus_masked-fvb4-2-processed-gene-107.10	cis	166527457	0.18	0.0202	100%	0.0	0.0	1.2	1.0	0.1	RGA1_SOLBU disease resistance RGA1 RGA3-blb
augustus_masked-fvb4-2-processed-gene-258.8	cis	166505336	0.35	0.0015	25.1%	4.6	0.0	1.4	1.3	0.1	LRX2_ARATH Leucine-rich repeat extensin 2
augustus_masked-fvb5-1-processed-gene-238.7	cis	123365994	0.09	0.0012	74.1%	0.8	0.8	1.4	2.8	0.5	HSL1_ARATH Receptor kinase HSL1 HAESA-LIKE1
augustus_masked-fvb5-1-processed-gene-71.8	cis	166524323	0.48	0.0080	35.5%	1.7	0.0	10.3	4.4	3.0	TIR_ARATH Toll interleukin-1 receptor
augustus_masked-fvb5-3-processed-gene-135.4	cis	166523635	0.26	0.0124	63.7%	0.5	0.2	0.1	0.0	0.0	MKKA_DICDI Mitogen-activated kinase kinase
augustus_masked-fvb5-4-processed-gene-18.1	cis	166518037	0.35	0.0110	26.6%	0.8	1.4	0.7	2.5	0.8	RGA1_SOLBU disease resistance RGA1 RGA3-blb
augustus_masked-fvb5-4-processed-gene-241.6	cis	123525092	0.44	0.0007	64.9%	1.4	0.1	2.5	2.1	1.8	LRX2_ARATH Leucine-rich repeat extensin 2 2 LRR
augustus_masked-fvb6-1-processed-gene-345.10	cis	166524541	0.21	0.0050	31.7%	1.8	0.7	4.1	4.1	2.2	HSL1_ARATH Receptor kinase HSL1 HAESA-LIKE1
augustus_masked-fvb7-1-processed-gene-57.2	cis	166509572	0.12	0.0003	73.3%	0.5	0.2	0.6	0.2	13.2	TIR_ARATH Toll interleukin-1 receptor
augustus_masked-fvb7-2-processed-gene-302.13	cis	166517211	0.29	0.0083	69.2%	2.3	0.0	2.1	0.1	0.0	MKKA_DICDI Mitogen-activated kinase kinase
augustus_masked-fvb7-2-processed-gene-53.6	cis	166509530	0.49	0.0118	35.7%	1.2	0.2	0.5	0.4	2.2	RGA1_SOLBU disease resistance RGA1 RGA3-blb
augustus_masked-fvb7-2-processed-gene-54.1	cis	166509530	0.49	0.0121	100%	9.9	0.1	0.4	0.3	11.5	LRX2_ARATH Leucine-rich repeat extensin 2 2 LRR
maker-fvb1-4-augustus-gene-30.48	cis	123365069	0.33	0.0175	63.8%	2.5	0.4	3.1	4.2	2.5	HSL1_ARATH Receptor kinase HSL1 HAESA-LIKE1
maker-fvb2-1-augustus-gene-182.42	cis	89877559	0.46	0.0225	80.9%	2.1	3.8	2.2	3.2	0.8	MKKA_DICDI Mitogen-activated kinase
maker-fvb2-1-snap-gene-111.27	cis	166503168	0.29	0.0002	100%	0.5	0.0	0.0	2.0	1.2	RGA1_SOLBU disease resistance RGA1 RGA3-blb
maker-fvb7-1-snap-gene-223.45	cis	123540423	0.45	0.0077	70.3%	2.4	0.1	0.1	0.0	1.7	LRX2_ARATH Leucine-rich repeat extensin 2 2 LRR
maker-fvb7-4-snap-gene-48.49	cis	166508582	0.34	0.0357	75.1%	0.3	0.4	0.1	0.0	0.2	HSL1_ARATH Receptor kinase HSL1 HAESA-LIKE1
maker-fvb7-4-snap-gene-59.59	cis	166518351	0.50	0.0064	4.7%	0.9	0.4	1.8	0.1	1.6	TIR_ARATH Toll interleukin-1 receptor
maker-fvb7-4-snap-gene-59.63	cis	166518351	0.50	0.0092	44.8%	0.1	0.0	0.6	0.0	0.4	MKKA_DICDI Mitogen-activated kinase
maker-fvb7-4-snap-gene-69.51	cis	123359450	0.17	0.0002	82.6%	2.3	1.8	2.1	3.6	2.2	RGA1_SOLBU disease resistance RGA1 RGA3-blb
snap_masked-fvb1-2-processed-gene-79.33	cis	123359751	0.08	0.0068	98.3%	0.0	0.0	0.0	0.0	2.0	LRX2_ARATH Leucine-rich repeat extensin 2 2 LRR
snap_masked-fvb2-1-processed-gene-107.14	cis	89780995	0.22	0.0011	75.1%	2.1	2.6	8.5	4.0	3.4	HSL1_ARATH Receptor kinase HSL1 HAESA-LIKE1
snap_masked-fvb3-2-processed-gene-11.25	cis	166509770	0.37	0.0084	11.4%	0.2	0.2	1.1	1.5	0.7	TIR_ARATH Toll interleukin-1 receptor
snap_masked-fvb3-3-processed-gene-288.15	cis	123361033	0.45	0.0095	98.9%	0.9	0.5	2.0	1.4	1.3	MKKA_DICDI Mitogen-activated kinase kinase kinase A
snap_masked-fvb6-1-processed-gene-37.31	cis	166519417	0.34	0.0411	24.2%	13.3	19.2	0.9	0.5	0.0	RGA1_SOLBU disease resistance RGA1
snap_masked-fvb7-2-processed-gene-254.35	cis	123357141	0.33	0.0011	36.5%	0.0	0.0	0.0	0.0	0.0	LRX2_ARATH Leucine-rich repeat extensin 2 2
maker-fvb7-1-snap-gene-273.51	cis	166508667	0.43	0.0319	35.7%	0.0	0.0	0.0	0.4	0.0	Y3475_ARATH LRR receptor-like serine threonine- kinase
maker-fvb3-4-augustus-gene-265.40	cis	166513103	0.13	0.0007	54.7%	4.4	5.3	2.6	18.8	5.2	GLO5_ARATH Peroxisomal(S)-2-hydroxy-acid oxidase GLO5
maker-fvb5-1-snap-gene-191.37	cis	166523649	0.13	0.0000	36.5%	0.0	0.0	0.0	0.0	0.1	RPM1_ARATH Disease resistance RPM1
maker-fvb5-2-augustus-gene-59.20	cis	123364094	0.46	0.0036	46.0%	0.0	0.3	4.0	12.0	0.8	probable LRR receptor-like serine threonine- kinase At5g48740
maker-fvb5-2-augustus-gene-61.13	cis	123364094	0.46	0.0014	71.7%	0.0	0.3	2.2	1.6	0.8	P2B10_ARATH F-box PP2-B10 PHLOEM PROTEIN 2-LIKE
maker-fvb5-2-augustus-gene-63.17	cis	123364094	0.46	0.0072	16.5%	0.0	0.0	3.5	3.7	1.1	P2B11_ARATH F-box PP2-B11 PHLOEM PROTEIN 2-LIKE
maker-fvb5-2-snap-gene-4.75	cis	166506813	0.32	0.0000	29.9%	2.1	9.3	37.2	43.0	1.4	DRL28_ARATH Probable disease resistance At4g27220
maker-fvb5-2-snap-gene-61.17	cis	123364094	0.46	0.0291	78.4%	0.0	0.2	1.3	1.4	0.3	P2B11_ARATH F-box PP2-B11 PHLOEM PROTEIN 2-LIKE
maker-fvb5-3-augustus-gene-135.25	cis	89832439	0.37	0.0040	11.5%	0.0	0.0	1.6	0.3	1.3	RPM1_ARATH Disease resistance RPM1
maker-fvb4-3-snap-gene-155.68	cis	123524810	0.21	0.0016	100%	0.1	0.2	0.6	0.1	0.5	TMVRN_NICGU TMV resistance N
maker-fvb5-3-snap-gene-221.67	cis	89893608	0.11	0.0045	94.4%	0.5	0.2	0.1	1.6	0.1	TMVRN_NICGU TMV resistance N
maker-fvb5-3-snap-gene-254.50	cis	166523796	0.21	0.0155	51.8%	0.1	0.1	0.4	0.5	0.1	TMVRN_NICGU TMV resistance N
maker-fvb5-4-snap-gene-125.42	cis	166506186	0.18	0.0078	93.2%	0.6	0.0	2.0	2.3	1.4	RGA3_SOLBU RGA3 Blight resistance B149
maker-fvb6-1-augustus-gene-153.32	cis	166507404	0.17	0.0000	76.3%	0.0	0.0	23.5	6.8	17.5	RGA3_SOLBU RGA3 Blight resistance B149
maker-fvb5-2-augustus-gene-61.14	cis	123358673	0.47	0.0167	44.9%	0.0	0.1	0.7	1.2	0.3	P2B10_ARATH F-box PP2-B10 PHLOEM PROTEIN 2-LIKE
augustus_masked-fvb7-1-processed-gene-284.2	cis	123359573	0.4	0.0139	95.1%	0.3	0	0.1	0.3	0.2	EMS1_ARATH Leucine-rich repeat receptor kinase EMS1
snap_masked-fvb6-2-processed-gene-263.31	cis	89781514	0.24	0.0084	54.9%	0	0	4.3	4.3	2.2	TMVRN_NICGU TMV resistance N
maker-fvb6-1-augustus-gene-160.45	cis	166515747	0.21	0.0340	38.4%	12.6	8.8	11.9	4.2	10.3	DGK5_ARATH Diacylglycerol kinase 5

Genetic association results for 61 transcriptomes are shown, detailing *cis* genetic factors controlling differentially expressed R-genes. The most significant marker name, minor allele frequency, FDR-adjusted p-value, narrow sense heritability, transcript accumulation in cultivars, and BLAST2GO description are shown.

**Table 3:** *Cis* and *trans* eQTL pertaining to fruit-expressed genes in *F. ×ananassa*

R-gene Name	eQTL phase	IStraw35 AX-	MAF	*p*-value (FDR)	h^2^ estimate	Mara	Elya-na	Radi-ance	Festi-val	Winter dawn	Description
maker-fvb4-2-snap-gene-5.61	cis	166505436	0.38	0.0214	34.3%	1.5	0.9	1.6	3.0	2.3	PSKR1_ARATH LRR receptor kinase 1 serine-threonine kinase
	trans	123361503	0.38	0.0214							
maker-fvb5-1-augustus-gene-139.47	cis	123524951	0.28	0.0482	77.5%	0.3	0.4	5.8	1.5	1.0	PIRL5_ORYSJ Plant intracellular Ras-group-related LRR 5
	trans	123361742	0.14	0.0482							
maker-fvb5-1-augustus-gene-263.34	cis	123357041	0.18	0.0415	14.6%	8.6	4.0	3.3	3.7	3.2	R13L1_ARATH disease resistance RPP13 1
	trans	166506561	0.18	0.0415							
maker-fvb5-2-snap-gene-213.46	cis	89817904	0.10	0.0006	79.8%	1.7	1.6	3.3	0.0	1.4	DRL21_ARATH disease resistance At3g14460
	trans	123539826	0.10	0.0006							
maker-fvb5-2-snap-gene-46.64	cis	166506808	0.48	0.0192	67.1%	3.4	0.0	1.3	1.0	1.4	GSO1_ARATH LRR receptor-like serine threonine- kinase GSO1
	trans	166522785	0.48	0.0192							
maker-fvb5-3-snap-gene-244.48	cis	123367068	0.48	0.0046	39.5%	0.4	0.5	4.7	5.6	2.8	Y4265_ARATH Probable LRR receptor-like serine threonine- kinase
	trans	166524268	0.48	0.0046							
maker-fvb5-4-snap-gene-110.38	cis	166523511	0.21	0.0001	100%	1.0	1.3	0.4	1.6	0.3	PIRL5_ORYSJ Plant intracellular Ras-group-related LRR 5
	trans	166524147	0.29	0.0014							
maker-fvb6-1-augustus-gene-27.58	cis	123363787	0.38	0.0028	91.2%	0.3	0.0	0.2	0.5	0.0	DRL42_ARATH Probable disease resistance At5g66900
	trans	123358884	0.38	0.0028							
maker-fvb6-3-snap-gene-412.71	cis	123614270	0.27	0.0457	74.4%	1.8	1.1	1.4	2.1	0.9	TIR_ARATH Toll interleukin-1 receptor
	trans	166525307	0.27	0.0457							
augustus_masked-fvb6-2-processed-gene-268.10	cis	166525890	0.48	0.0021	91.2%	0.8	0.8	0.3	1.7	1.2	HSL1_ARATH Receptor kinase HSL1 HAESA-LIKE1
	trans	123362183	0.48	0.0021							
augustus_masked-fvb6-3-processed-gene-176.9	cis	166515622	0.33	0.0004	65.6%	0.4	0.0	7.9	20.0	0.1	ADT3_ARATH ADP,ATP carrier mitochondrial ADP ATP
	trans	123525691	0.33	0.0005							
augustus_masked-fvb7-2-processed-gene-63.0	cis	166508452	0.30	0.0047	27.7%	0.5	0.1	1.3	0.1	0.0	TMVRN_NICGU TMV resistance N
	trans	89823698	0.28	0.0047							
augustus_masked-fvb7-3-processed-gene-243.6	cis	166517344	0.38	0.0204	66.5%	47.5	14.1	0.1	8.1	0.7	DR100_ARATH DNA damage-repair toleration DRT100
	trans	89894427	0.38	0.0204							
maker-fvb1-2-snap-gene-63.40	cis	166510935	0.17	0.0015	86.2%	0.5	0.1	1.3	0.1	0.0	TMVRN_NICGU TMV resistance N
	trans	166525497	0.16	0.0015							
maker-fvb1-2-snap-gene-96.40	cis	166517617	0.18	0.0020	67.6%	0.0	0.0	1.0	0.0	0.4	DRL42_ARATH Probable disease resistance
	trans	166525528	0.18	0.0020							
maker-fvb1-4-snap-gene-66.72	cis	123363545	0.28	0.0280	68.4%	3.9	0.2	12.0	12.0	7.4	U496I_ARATH UPF0496 At2g18630
	trans	166516240	0.18	0.0093							
maker-fvb1-4-snap-gene-76.45	cis	123357162	0.18	0.0001	100%	0.7	0.2	0.0	0.0	0.0	DRL43_ARATH Probable disease resistance
	trans	166516240	0.18	0.0001							
maker-fvb7-2-augustus-gene-136.47	cis	166526312	0.43	0.0116	79.9%	0.3	0.0	0.4	0.4	0.2	TMVRN_NICGU TMV resistance N
	trans	123359434	0.46	0.0371							
maker-fvb7-2-augustus-gene-147.47	cis	123359385	0.45	0.0003	45.2%	1.1	0.2	5.5	3.1	2.1	TMVRN_NICGU TMV resistance N
	trans	123365359	0.42	0.0011							
maker-fvb7-2-augustus-gene-163.44	cis	123364494	0.39	0.0011	52.3%	0.7	0.3	2.0	0.9	0.8	RGA3_SOLBU disease resistance RGA3 Blight resistance B149
	trans	123365359	0.42	0.0055							
maker-fvb7-2-augustus-gene-65.21	cis	166512110	0.21	0.0031	69.2%	5.3	0.3	0.9	1.2	0.5	RP8L2_ARATH Probable disease resistance RPP8 2
	trans	166509598	0.21	0.0031							
maker-fvb7-2-snap-gene-161.50	cis	123364494	0.39	0.0052	38.9%	2.4	1.0	4.0	2.0	2.6	MAP1A_ARATH Methionine aminopeptidase 1A MAP 1A 1A
	trans	123365359	0.42	0.0154							
snap_masked-fvb3-4-processed-gene-8.19	cis	166521734	0.16	0.0023	69.6%	2.2	4.3	6.2	5.2	4.0	TMVRN_NICGU TMV resistance N
	trans	89826525	0.17	0.0033							
snap_masked-fvb6-1-processed-gene-352.19	cis	123366334	0.27	0.0306	76.8%	0.1	0.0	0.8	1.1	0.8	TMVRN_NICGU TMV resistance N
	trans	123357007	0.42	0.0465							

Genetic association results for 61 transcriptomes are shown, detailing *cis* and *trans* genetic factors controlling differentially expressed R-genes. The most significant marker name, minor allele frequency, FDR-adjusted *p*-value, narrow sense heritability, transcript accumulation in cultivars, and BLAST2GO description are shown.

**Figure 4:**
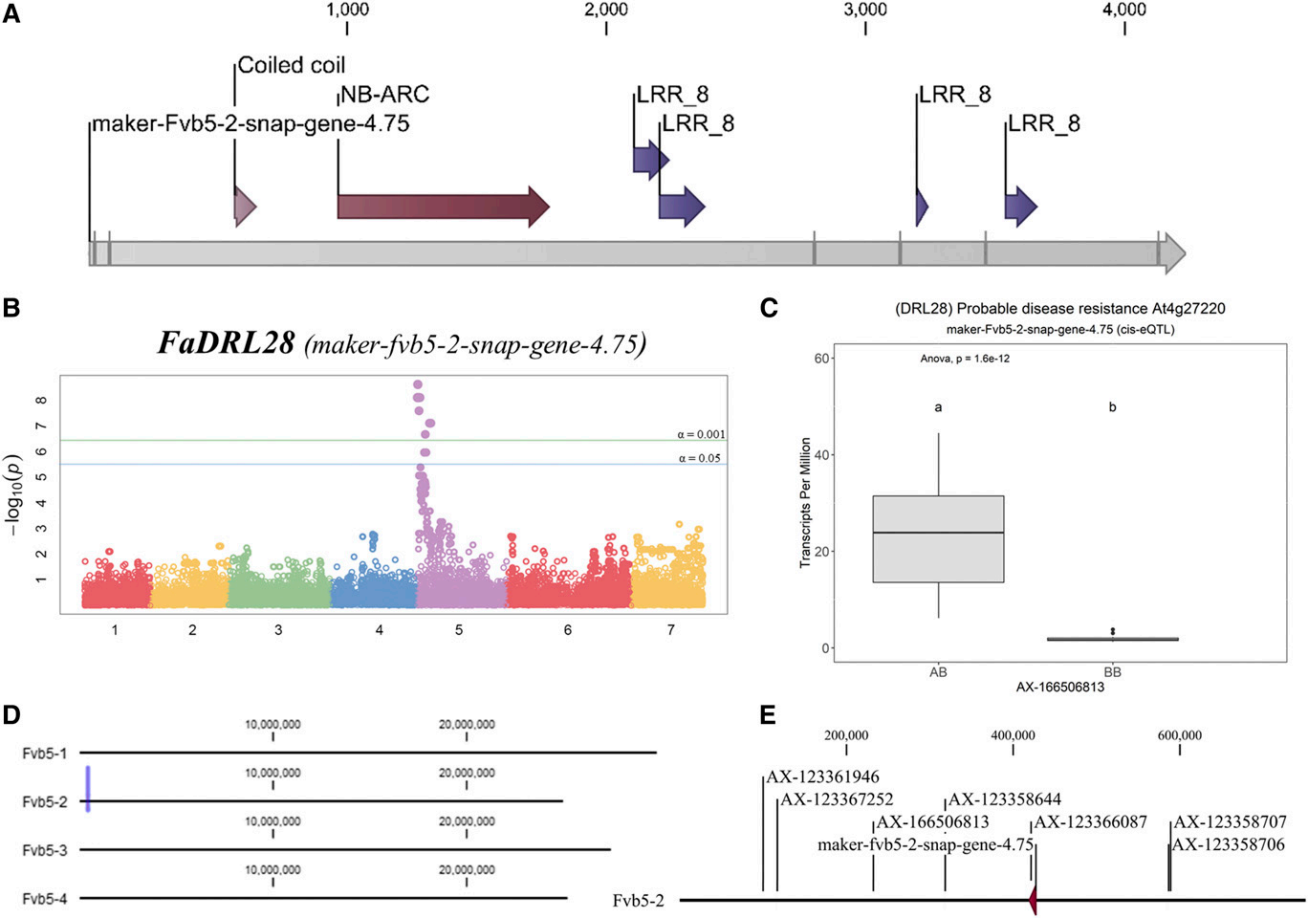
Example *cis*-eQTL of a Fruit-Expressed Strawberry R-gene. A. Domain analysis of the ‘Camarosa’ putative resistance gene *FaDRL28*, a CC-NBS-LRR R-gene. Gray lines delineate exon-exon borders in the predicted mature transcript. B. Octoploid fruit expression of *FaDRL28* associates with a single locus on chromosome 5. C. A single dose of an “A” allele increases mean transcription of *FaDRL28* to above 25 TPM (“AB” genotype) from a mean approximately 1 TPM (“BB” genotype) (single-marker ANOVA *p*-value 1.6E-12). D. The *Fvb5-2* subgenomic location of *FaDRL28* in the octoploid ‘Camarosa’ genome is indicated (purple vertical line). E. Seven equally-significant eQTL markers (*p*-value 2.73E-05, post-FDR adjustment) show close subgenomic co-localization with *FaDRL28*, including one marker within the *FaDRL28* coding sequence (AX-123366087).

A representative eQTL R-gene (maker-fvb5-2-snap-gene-4.75) is detailed in Figure 4. Analysis by BLAST2GO indicates a probable disease resistance gene homologous to the *Arabidopsis thaliana* gene At4g27220 with the Uniprot identifier DRL28_ARATH. This gene is hereafter referred to as “*FaDRL28*”. A CC-NBS-NLR structure is predicted for *FaDRL28* (Figure 4A). An eQTL was detected for this gene relative to chromosome 5 on the *F. vesca* genome position (Figure 4B). This eQTL is associated with increased transcription of *FaDRL28* from near-zero to above 25 TPM (single-marker ANOVA *p*-value 1.6E-12) (Figure 4C). This eQTL is superficially analogous to the physical position of octoploid *FaDRL28* on chromosome 5, homeolog 2 (Figure 4D). The significant markers are not included in the ‘Holiday’ × ‘Korona’ octoploid genetic map, impeding a recombination-based subgenomic genetic association (van Dijk *et al.* 2014). However, the associated marker physical sequences match fairly uniquely to the ‘Camarosa’ chromosome 5-2 subgenomic locus, confirming a *cis*-eQTL designation (Figure 4E). One eQTL marker locates inside the *FaDRL28* coding sequence.

### Evolutionary pressure on F. ×ananassa R-genes

**Figure 5:**
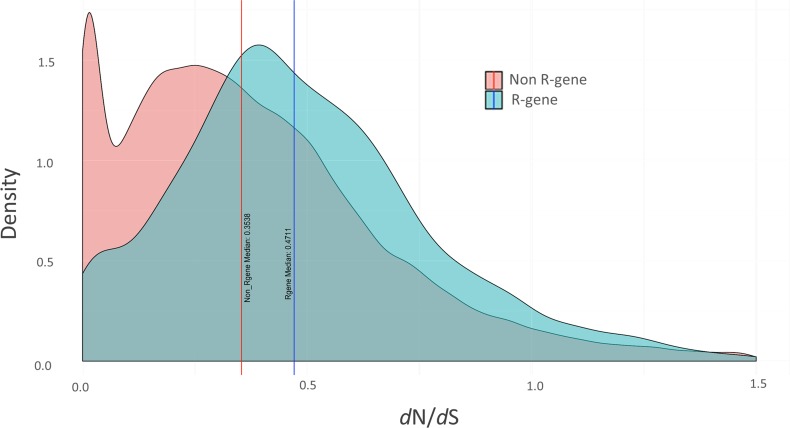
Evolutionary Pressures on *F*. × *ananassa* R-genes. The median *d*N/*d*S ratio for R-genes (0.47) is higher than for non R-genes (0.35). Density curves for *F*. **×***ananassa* R-genes (blue) and non R-genes (red) are calculated based on comparison to the closest ancestral diploid homolog from *F. vesca*.

Elevated median *d*N/*d*S ratios were observed across all 1,962 predicted *F*. ***×*** *ananassa* R-genes (0.47) compared to non R-genes (0.35) (Figure 5). Fewer R-genes exhibited extremely low *d*N/*d*S ratios, indicating that high degrees of R-gene conservation are less common. However, a similar rate of hypervariable genes (*d*N>>*d*S) was observed between R-genes and non R-genes. Median *d*N/*d*S values for RPW8-type R-genes (0.62) are significantly higher than for general R-genes (0.47) as confirmed by one-way ANOVA [F (1, 1911) = 10.6, *P* < 0.0012] (Table S1). A complete list of dN/*d*S ratios for each *F*. ***×****ananassa* R-gene is provided in Table S1.

R-gene *d*N/*d*S values were compared against transcript accumulation across various strawberry tissues and receptacle stages. R-genes with low transcript accumulation across all tissues were correlated with higher *d*N/*d*S ratios (Pearson’s *r* = -0.69, *P* < 0.0001) (Figure S5). In other words, R-genes with poor evidence of expression also have higher ratios of non-synonymous mutation capable of altering amino acid sequences and affecting protein function.

### Subgenome dominance in octoploid strawberry

**Figure 6:**
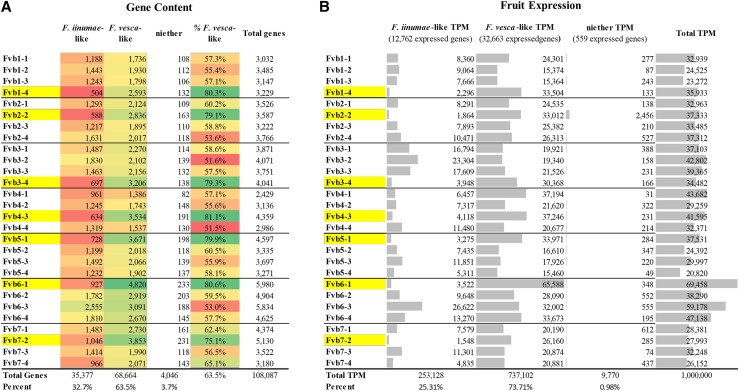
General Retention and Expression Bias in Octoploid Strawberry. ‘Camarosa’ gene models from every chromosome are categorized as either more *F. vesca*-like, more *F. iinumae*-like, or neither. Red-green color scale indicates low-to-high gene content, respectively. Yellow highlight indicates the most *F. vesca*-like homeologous chromosome. A. Gene content per homeologous chromosome, by putative ancestral gene similarity. B. Relative transcript accumulation of all genes in the fruit, by putative ancestral similarity.

Polyploidization is associated with rapid genome remodeling events to establish a new homeostasis, including selective gene loss and methylation. While R-gene expansiveness is often considered evolutionarily favorable, genes that are stoichiometrically or dosage sensitive are more commonly retained in duplicate after polyploidization (Edger and Pires 2009; Birchler and Veitia 2012; Edger *et al.* 2017a). The ‘Camarosa’ octoploid genome, in comparison with the genomes from its diploid *F. vesca*-like and *F. iinumae*-like ancestors, has provided an ideal platform to study the general biological phenomena of post-hybridization genome remodeling and subgenome dominance (Edger *et al.* 2019). To gauge R-gene post-hybridization retention specifically, a gene-focused baseline assessment of subgenome dominance in the ‘Camarosa’ octoploid genome was necessary. Putative gene ancestry was predicted based on gene-by-gene sequence comparisons to determine the closest ‘Camarosa’ gene homologs in *F. vesca* (Fragaria_vesca_v2.0.a2.cds) and *F. iinumae* (FII_r1.1cds), which is representative of the highly similar ‘old world’ subgenomes. This gene-by-gene putative orthology analysis was selected over a total comparison of homeologous chromosomes, as extensive genetic transfer from the *F. vesca*-like subgenome has strongly converted all subgenomes to contain *F. vesca*-like genes over time (Tennessen *et al.* 2014), and because the *F. iinumae* FII_r1.1 genome is incompletely assembled and is not amenable to whole-genome alignment. By this facile coding-sequence comparison method, a significant bias toward the retention and/or expansion of *F. vesca*-like genes is observed in the ‘Camarosa’ genome (Figure 6A), with an even stronger bias toward *F. vesca*-like fruit gene expression (Figure 6B) consistent with previous analyses (Edger *et al.* 2019). Of 108,087 *F. ×ananassa* ‘Camarosa’ predicted gene models, 68,664 genes (63.5%) were most similar to an *F. vesca* gene model, with 35,377 (32.7%) most similar to an *F. iinumae* gene model, with a minority of genes not closely matching either. A single homeologous chromosome with significantly more *F. vesca*-like genes (∼80% *F. vesca*-like) was seen in every chromosomal group. In a majority of cases, this putative *F. vesca*-derived chromosome possesses the greatest total gene content of the chromosome group. Gene expression-based subgenome bias was assessed using mature fruit transcriptomes averaged from the cultivars ‘Florida Elyana’, ‘Mara de Bois’, ‘Florida Radiance’, ‘Mara des Bois’, ‘Strawberry Festival’, and ‘Winter Dawn’. In these cultivars, 73.7% of total transcripts derived from a gene sequence most similar to *F. vesca*, corresponding to a 10.2% expression increase relative to the baseline genomic retention bias. This bias toward the expression of *F. vesca*-like sequences was seen on every subgenome (Figure 6B, yellow highlight).

### NLR-gene subgenome dominance

**Figure 7:**
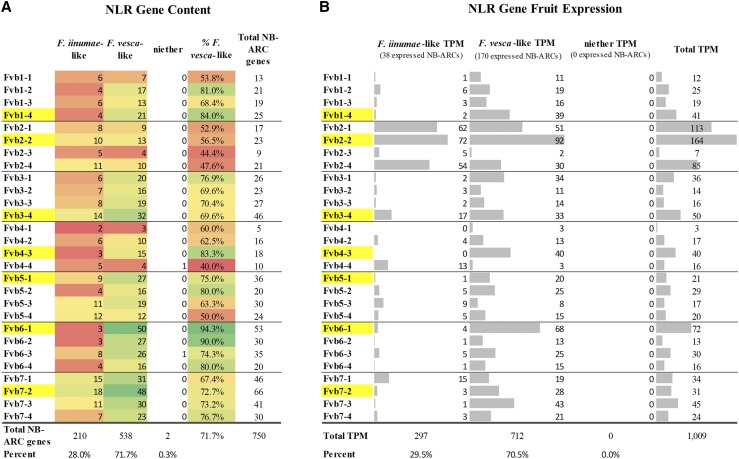
NLR-gene Retention and Expression Bias in Octoploid Strawberry. ‘Camarosa’ NB-ARC domain containing-NLR models from every chromosome are categorized as either more *F. vesca*-like, more *F. iinumae*-like, or neither. Red-green color scale indicates low-to-high gene content, respectively. Yellow highlight indicates the most F. vesca-like homeologous chromosome. A. NLR-gene content per homeologous chromosome, by putative ancestral gene similarity. B. Relative transcript accumulation of NLR-genes in the fruit, by putative ancestral similarity.

Significant gene retention bias toward NLR genes that are more *F. vesca*-like is observed in ‘Camarosa’ gene models (Figure 7). Of the 750 predicted NLR-gene models containing an NB-ARC domain, 69.3% more closely resemble a *F. vesca* gene rather than an *F. iinumae* gene (Figure 7A). This is somewhat higher than the baseline retention bias toward *F. vesca*-like genes in octoploid (63.8%) from this analysis. In every chromosome group, the *F. vesca*-like homeologous chromosomes (yellow highlight) retained the greatest number of NB-ARC domain containing-NLRs. Overall, 538 NB-ARC domain containing-NLRs demonstrate the highest sequence identity with an *F. vesca* gene, 210 show highest sequence identity with an *F. iinumae* gene, and 2 (an RPW8-only gene, and an LRR_8-only gene) are without significant matches to either diploid genome. While *F. vesca*-like genes contribute the most to total NLR expression across 61 mature fruit transcriptomes (70.5% of transcripts), this is proportional to *F. vesca*-like NLR genome content (71.3%) and is similar in magnitude to general *F. vesca* expression bias (73.7%) (Figure 7B). In other words, *F. vesca*-like NLR genes are retained in the octoploid genome somewhat above the baseline bias, but do not experience the additional expression magnitude bias that is a generic feature of *F. vesca*-like transcripts. A greater breadth of genome-predicted NLR genes had evidence of expression across mature fruit transcriptomes (246 genes, n = 61) (Figure 7B) than in the ‘Camarosa’ mature fruit transcriptome alone (139 genes, n = 5) (Figure 3B) however averaged transcriptional magnitude was similar (Figure 3C).

### RenSeq for strawberry resistance genes

**Figure 8:**
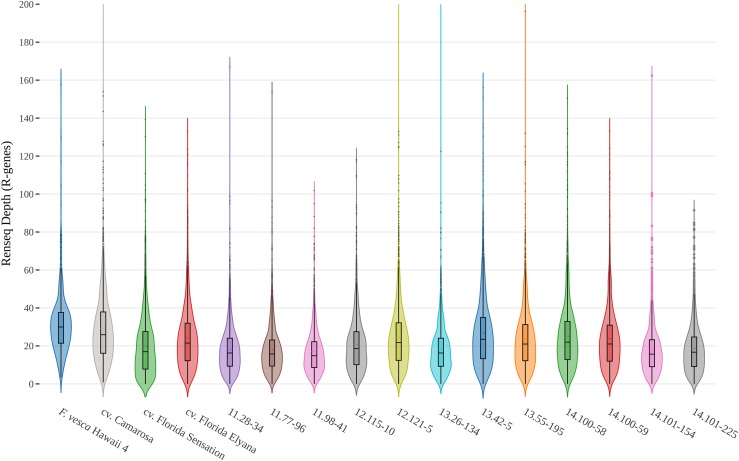
RenSeq Increases Sequencing Depth for R-gene Loci in Multiplexed Octoploid Genomes. Sixteen disease-resistant strawberry genomic libraries (fifteen octoploid accessions and diploid F. vesca) were enriched for R-genes and sequenced via Illumina HiSeq yielding an average of 2.60 million reads per genomic library. Violin plots indicate the range of R-gene resequencing depth from each genomic RenSeq library. Roughly half of all sequencing reads mapped to a previously-identified R-gene locus, representing a substantial sequence enrichment relative to R-gene genomic representation.

A panel of sequence capture probes was designed based on putative R-gene sequences discovered in the genomes of *F. ×ananassa* ‘Camarosa’, *F. vesca* genotype Hawaii 4, *F. iinumae*, and *de novo* fruit transcriptomes from *F. ×ananassa* cultivars ‘Mara des Bois’ and ‘Florida Elyana’. Benchtop RenSeq capture on genomic DNA was performed on a collection of sixteen strawberry genotypes, including twelve *F. ×ananassa* advanced breeding selections, three *F. ×ananassa* disease-resistant cultivars, and a diploid *F. vesca*. As a preliminary validation of capture efficiency with this novel RenSeq probe panel, multiplexed Illumina sequencing was performed on captured R-gene genomic libraries. An average of 2.60 million reads (2 × 100bp) was obtained for each of sixteen libraries from a single lane. Reads from octoploid and diploid lines were mapped to their respective annotated genomic references. An average R-gene resequencing depth of 26x was achieved in the ‘Camarosa’ RenSeq line and 30x in the *F. vesca*, with similar coverage ranges in the other diverse octoploid accessions (Figure 8). In the ‘Camarosa’ RenSeq line, 68% of reads mapped to an annotated resistance gene, while an additional 20% of reads mapped to a non-R-gene gene model. In *F. vesca* this efficiency was lower, where 36% of reads mapped to an annotated R-gene. A FASTA of RenSeq probes is provided for use in File S2. Example probe coverage is detailed in Figure S3.

## Discussion

These results provide a characterization of the R-gene complement of cultivated octoploid strawberry and the relationship to the extant diploid relatives, *F. vesca* and *F. iinumae*. Commercial strawberry is hypothesized to contain a single *F. vesca*-like subgenome, and three highly-similar ‘old world’ subgenomes which are likely derived from *F. iinumae*, *F. viridis*, and *F. nipponica* (Edger *et al.* 2019). Polyploidization is associated with massive genome remodeling events including gene loss (Edger *et al.* 2018; Edger *et al.* 2017a). Linkage-map comparisons in octoploid and diploid strawberry have uncovered extensive unidirectional homeologous exchanges which have broadly converted the three ‘*old world’ F. innumae*-like subgenomes to be more *F. vesca*-like (Tennessen *et al.* 2014). This finding explains the difficulties of clear ancestral delineation of strawberry homeologs (Vining *et al.* 2017). Recent analysis of the octoploid genome reveals that biased homologous exchanges have converted other subgenomes to be more like the dominant *F. vesca*-like subgenome (Edger *et al.* 2019). The present gene-level homology and expression analysis shows the majority of *F. vesca*-like dominance is derived from *F. vesca*-like genes residing on alternate subgenomes. For NLR genes in particular, the bias toward *F. vesca*-like genomic retention was more pronounced. Unlike general octoploid genes, expression of *F. vesca*-like and *F. iinumae*-like NLRs is proportional to their genomic representation. This finding provides potential insight into the practical drivers of subgenome conversion. Consolidation of redundant genes and maintenance of stoichiometrically sensitive genes has been hypothesized as a driver for gene retention bias (Edger and Pires 2009; Birchler and Veitia 2012; Edger *et al.* 2017a). NLRs are involved in consequential and sensitive protein-level interactions, including signaling functions requiring homo- and hetero-dimerizations (El Kasmi and Nishimura 2016). Avoidance of dysfunctional NLR molecular interactions may have contributed to the observed biases in NLR retention and expression, post-polyploidization.

Multiple distinct NLR clades with identical domain architectures were detected, likely distinguishing intra-subgenome homologs from different subgenomes. These likely reflect broad ancestral sequence divergences prior to hybridization. Comparison of R-genes in octoploid and diploid strawberry reveals enrichment of different subtypes. The ‘Camarosa’ genome shows a large increase in complete TNL-type R-genes and a concomitant decrease in truncated TIR-only genes, relative to its diploid ancestral relatives. The *F. iinumae* genome shows a considerably larger amount of CNL-types. TNLs have been nearly eliminated from most monocot genomes in bias toward CNL-types (Nepal *et al.* 2017). The reasons for emerging divisions in TNL/CNL content in plant genomes remains unclear. In hybrid *F*. ×*ananassa*, it is possible that relatively high number of complete TNL genes is a result of higher rate of retention post-polyploidization in this category. Many of the non-classical domains discovered in ‘Camarosa’ R-genes have also been found and characterized in the R-genes of other species. These include an LRR/Malectin-like RLK protein, which mediates powdery mildew resistance in barley and wheat (Rajaraman *et al.* 2016). Atypical R-gene domains physically associated with NB-ARCs have been implicated in a variety of active disease resistance functions, including signal transduction and defense gene activation, and serving as decoy endogenous sequences to bait pathogen effectors into direct interaction and detection (Khan *et al.* 2016).

A large proportion of strawberry NLR genes from octoploid and diploid genomes are associated with RPW8 domains. It has been suggested that the RPW8 domain emerged with the earliest land plants and subsequently merged with NLR genes, however their prevalence across plant genomes varies widely (Zhong and Cheng 2016). The RPW8 domain appears to have been completely lost in monocots, and is rare in many other species. R-gene genomic studies frequently neglect to assess the presence of NLR-associated RPW8 domains. Two NBS-RPW8 proteins conferring mildew-resistance have been described in the *Arabidopsis thaliana* genome (Xiao *et al.* 2001) that retained their function when expressed in grape (Hu *et al.* 2018). The *At*RPW8.2 gene was recently shown to induce the expression of defense-related genes when expressed in strawberry leaves (Cui *et al.* 2017). This R-gene subtype has apparently expanded in strawberry, possibly due to unusually high mildew disease pressure exerted on strawberry species and intense selection for resistance. However, R-gene domain content is not reliably predictive of resistance specificity, and close R-gene paralogs are known to confer resistance to pathogens in entirely different kingdoms (Wen *et al.* 2015). Interestingly, the strawberry RPW8 domain is frequently found in association with NB-ARC-containing genes but never with TIRs. RPW8-containing genes are similar to general R-genes in terms of their frequency of expression, eQTL discovery rate, and putative orthology with *F. vesca* (Table S1). However, a significant and large difference was noted in terms of *d*N/*d*S ratio, indicating a higher degree of protein-level variability for this subclass. The purpose of RPW8 gene diversification and expansion in strawberry remains an interesting open question.

Octoploid NLR transcript accumulation is low throughout the strawberry plant, but is particularly low in the mature receptacle. This is an unexpected result due to the many pathogens targeting this susceptible organ. It is possible that only certain R-genes are highly upregulated in the response to pathogen attack. Another possibility is that resistance based on the hypersensitive response may be less effective at mature stages, where cell wall disruption has already initiated with ripening and the intercellular environment is conducive to pathogen growth. Transcriptional response to *Botrytis cinerea* infection in the mature octoploid receptacle led to differential expression of over 1,500 genes, including secondary metabolism and pathogenesis-related (PR) genes, but only 15 NLR genes (Xiong *et al.* 2018). In the present study, elevated NLR transcription in the green, white, and turning stages suggest NLR-based resistance may be more prevalent at these earlier developmental stages. The highest levels of NLR expression were seen in the roots and leaves, indicating this mode of resistance may be more common in these tissues. Root-dominant expression of NLRs is common in many but not all plant species (Munch *et al.* 2018). Strawberry NLR expression overlaps poorly between tissues, supporting the concept that NLRs are optimized for each tissue (Munch *et al.* 2018). Based on these patterns of expression, resistance to soil-borne pathogens via NLR-genes may be more common in strawberry. It would be interesting to examine the patterns of tissue-specific expression of R-genes against different strawberry pathogens, particularly the common soilborne pathogens causing strawberry verticillium wilt (*Verticillium dahlia*), charcol rot (*Macrophomina phaseolina*), and *Fusarium* wilt (*Fusarium oxysporum* f.sp. *fragariae*) (Zurn *et al.* 2018). Across 61 mature fruit transcriptomes, a greater number of putatively expressed NLR genes were found compared to ‘Camarosa’ mature fruit alone. This signifies possible genetic/environmental variabilities which can be measured via eQTL analysis.

The genetics of differential fruit expression of all R-genes in strawberry cultivars was examined via eQTL analysis. In many cases, the identified genetic markers described presence/absence of R-gene expression. The identified eQTLs were often due to a *cis* variant at a single detectable locus, very close to the physical position of the gene itself. This is suggestive of a mutation in a *cis*-regulatory element, such as the gene promoter or 5′-UTR, or a genic presence/absence structural variation. Such presence/absence variation affects nearly 20% of genes in the *Brassica oleracea* pangenome and is a major contributor of agronomic trait diversity (Golicz *et al.* 2016). As these strawberry R-gene eQTL are derived from crosses of cultivars with differing ranges of pathogen susceptibility, these eQTL genes represent strong candidates for functional disease resistance and potential genetic improvement. These disclosed R-gene eQTL marker sequences may be cross-referenced with existing disease-resistance QTL to potentially identify causal R-genes. As categories of R-genes are expressed at very low levels unless induced by pathogens (Lai and Eulgem 2017), the genotype × pathogen interaction may have lowered confidence values or introduced possible type II errors in eQTL detection. However, the reproducibility of *cis*-eQTL tends to be particularly high in related populations (Peirce *et al.* 2006). Additional replicates and infected/non-infected challenge conditions will likely reveal additional eQTL associations and greatly improve the confidence of heritability estimates, and may be used to validate pathogen-induced R-gene candidates.

*F*. ***×****ananassa* predicted R-genes (NLRs and other R-gene types) have elevated average *d*N/*d*S ratios compared to non R-genes, indicating greater overall tendency toward divergent selection. R-genes with very low *d*N/*d*S ratios are likely to be conserved disease resistance genes. This active evolutionary selection is highly indicative of function. Of particular interest are strawberry R-genes demonstrating both low *d*N/*d*S values and low transcript levels across all tissues (Table S1). Many functional R-genes are expressed at low levels, either constitutively or until elicited by the proper pathogen (Lai and Eulgem 2017). Such R-genes may be difficult to distinguish from pseudogenes on a purely transcriptional bases. Low *d*N/*d*S values demonstrate selective pressure to maintain these sequences, offering evidence of maintained function despite low expression. The results of this combinatorial analysis can be used help identify novel sources of R-gene-based resistance which may be otherwise difficult to detect. It should be noted that this analysis is performed in the context of a single cultivar, which has undergone several centuries of artificial selection. It is possible that wild octoploid species may reveal different and more natural patterns of disease-resistance selection. More sequenced accessions from geographically diverse wild and cultivated germplasm are needed. Further analysis on the octoploid pangenome will reveal more detailed selection patterns, and more importantly, reveal recent selection sweep events which may have occurred in certain R-gene groups.

Many R-genes were discovered clustered in the genomes of both octoploid and diploid strawberry, highlighting the challenges of resolving individual R-genes via association mapping and positional cloning. The difficulty of isolating functional R-genes from strawberry disease resistance QTL was the principle motivator of this analysis. A thorough identification of R-genes in the octoploid genome is necessary for future genomics and genetics analysis in strawberry disease-resistance breeding programs. Additionally, this information is prerequisite for creating a RenSeq probe panel, to facilitate targeted R-gene sequencing in breeding programs.

A novel strawberry RenSeq capture-probe library was developed based on the R-gene sequences identified from genomic and transcriptomic resources. This 39,501-probe panel was experimentally validated using octoploid and diploid genomes and resulted in an average ∼20× R-gene resequencing depth per genomic library, using only multiplexed short reads. RenSeq assembly in ‘Camarosa’ and the *F. vesca* genotype Hawaii 4 resulted in significant coverage of R-genes. Despite having the highest total resequencing depth, the capture efficiency in *F. vesca* (R-gene reads over total reads) was somewhat lower. It is possible that this represents the saturation of capture probes in a smaller genome. It is also possible that probes designed from non-*F. vesca*-like octoploid sequences bound spuriously in the *F. vesca* genome. Similar rates of perfect sequence matching along the entire read in ‘Camarosa’ and *F. vesca* (66.24% and 69.68%, respectively) indicates that theoretical octoploid reference sequence errors are not likely promoting RenSeq assembly error in ‘Camarosa’. However, 14.4% of mapped ‘Camarosa’ R-gene reads have an equally valid alternative R-gene mapping locus, compared with just 4.83% in *F. vesca*. This difference indicates that homeologous sequence redundancy is an appreciable issue for mapping short-reads in polyploids, even with an isogenic (but not haplotype-specific) mapping reference. Longer sequencing read-lengths, spanning less well-conserved non-coding sequences, will assist in *de novo* resolution of similar loci in octoploid strawberry. Combining RenSeq with long-read sequencing technologies will allow for improved *de novo* assembly of R-gene loci, and will greatly facilitate causal mutation detection within disease resistance QTL in octoploid strawberry.

## References

[bib1] Amil-Ruiz F., Blanco-Portales R., Muñoz-Blanco J., and Caballero J. L., 2011 The Strawberry Plant Defense Mechanism: A Molecular Review. Plant Cell Physiol. 52: 1873–1903. 10.1093/pcp/pcr136 21984602

[bib2] Anciro A., Mangandi J., Verma S., Peres N., Whitaker V.M., 2018 FaRCg1: a quantitative trait locus conferring resistance to Colletotrichum crown rot caused by Colletotrichum gloeosporioides in octoploid strawberry. Theoretical and Applied Genetics. 10.1007/s00122-018-3145-z 30032317

[bib3] Andolfo G., Jupe F., Witek K., Etherington G. J., Ercolano M. R., 2014 Defining the full tomato NB-LRR resistance gene repertoire using genomic and cDNA RenSeq. BMC Plant Biol. 14: 120 10.1186/1471-2229-14-120 24885638 PMC4036795

[bib4] Arya P., Kumar G., Acharya V., and Singh A. K., 2014 Genome-Wide Identification and Expression Analysis of NBS-Encoding Genes in Malus x domestica and Expansion of NBS Genes Family in Rosaceae. PLoS One 9: e107987 10.1371/journal.pone.0107987 25232838 PMC4169499

[bib5] Baumgartner I. O., Patocchi A., Frey J. E., Peil A., and Kellerhals M., 2015 Breeding Elite Lines of Apple Carrying Pyramided Homozygous Resistance Genes Against Apple Scab and Resistance Against Powdery Mildew and Fire Blight. Plant Mol. Biol. Report. 33: 1573–1583. 10.1007/s11105-015-0858-x

[bib6] Birchler J. A., and Veitia R. A., 2012 Gene balance hypothesis: Connecting issues of dosage sensitivity across biological disciplines. Proc. Natl. Acad. Sci. USA 109: 14746–14753. 10.1073/pnas.1207726109 22908297 PMC3443177

[bib7] Cockerton H. M., Vickerstaff R. J., Karlström A., Wilson F., Sobczyk M., 2018 Identification of powdery mildew resistance QTL in strawberry (Fragaria × ananassa). Theor. Appl. Genet. 131: 1995–2007. 10.1007/s00122-018-3128-0 29971472 PMC6096635

[bib8] Conesa A., Götz S., García-Gómez J. M., Terol J., Talón M., 2005 Blast2GO: a universal tool for annotation, visualization and analysis in functional genomics research. Bioinformatics 21: 3674–3676. 10.1093/bioinformatics/bti610 16081474

[bib9] Cordova L. G., Amiri A., and Peres N. A., 2017 Effectiveness of fungicide treatments following the Strawberry Advisory System for control of Botrytis fruit rot in Florida. Crop Prot. 100: 163–167. 10.1016/j.cropro.2017.07.002

[bib10] Cui M.-Y., Wei W., Gao K., Xie Y.-G., Guo Y., 2017 A rapid and efficient Agrobacterium-mediated transient gene expression system for strawberry leaves and the study of disease resistance proteins. Plant Cell Tissue Organ Cult. 131: 233–246 (PCTOC) 10.1007/s11240-017-1279-3

[bib11] Djian-Caporalino C., Palloix A., Fazari A., Marteu N., Barbary A., 2014 Pyramiding, alternating or mixing: comparative performances of deployment strategies of nematode resistance genes to promote plant resistance efficiency and durability. BMC Plant Biol. 14: 53 10.1186/1471-2229-14-53 24559060 PMC3944934

[bib12] Edgar R. C., 2004 MUSCLE: multiple sequence alignment with high accuracy and high throughput. Nucleic Acids Res. 32: 1792–1797. 10.1093/nar/gkh340 15034147 PMC390337

[bib13] Edger P. P., McKain M. R., Bird K. A., and VanBuren R., 2018 Subgenome assignment in allopolyploids: challenges and future directions. Curr. Opin. Plant Biol. 42: 76–80. 10.1016/j.pbi.2018.03.006 29649616

[bib14] Edger P. P., and Pires J. C., 2009 Gene and genome duplications: the impact of dosage-sensitivity on the fate of nuclear genes. Chromosome Res. 17: 699–717. 10.1007/s10577-009-9055-9 19802709

[bib15] Edger P. P., Poorten T. J., VanBuren R., Hardigan M. A., Colle M., 2019 Origin and evolution of the octoploid strawberry genome. Nat. Genet. 51: 541–547. 10.1038/s41588-019-0356-4 30804557 PMC6882729

[bib16] Edger P. P., Smith R., McKain M. R., Cooley A. M., Vallejo-Marin M., 2017 a Subgenome Dominance in an Interspecific Hybrid, Synthetic Allopolyploid, and a 140-Year-Old Naturally Established Neo-Allopolyploid Monkeyflower. Plant Cell 29: 2150–2167. 10.1105/tpc.17.00010 28814644 PMC5635986

[bib17] Edger P. P., VanBuren R., Colle M., Poorten T. J., Wai C. M., 2017 b Single-molecule sequencing and optical mapping yields an improved genome of woodland strawberry (Fragaria vesca) with chromosome-scale contiguity. Gigascience 7: 1–7. 10.1093/gigascience/gix124 PMC5801600 29253147

[bib18] El Kasmi F., and Nishimura M. T., 2016 Structural insights into plant NLR immune receptor function. Proc. Natl. Acad. Sci. USA 113: 12619–12621. 10.1073/pnas.1615933113 27803318 PMC5111699

[bib19] Farzaneh M., Kiani H., Sharifi R., Reisi M., and Hadian J., 2015 Chemical composition and antifungal effects of three species of Satureja (S. hortensis, S. spicigera, and S. khuzistanica) essential oils on the main pathogens of strawberry fruit. Postharvest Biol. Technol. 109: 145–151. 10.1016/j.postharvbio.2015.06.014

[bib20] Funk A., Galewski P., and McGrath J. M., 2018 Nucleotide-binding resistance gene signatures in sugar beet, insights from a new reference genome. Plant J. 95: 659–671. 10.1111/tpj.13977 29797366

[bib21] Golicz A. A., Bayer P. E., Barker G. C., Edger P. P., Kim H., 2016 The pangenome of an agronomically important crop plant Brassica oleracea. Nat. Commun. 7: 13390 10.1038/ncomms13390 27834372 PMC5114598

[bib22] Hammond-Kosack K. E., and Jones J. D. G., 1997 Plant Disease Resistance Genes. Annu. Rev. Plant Physiol. Plant Mol. Biol. 48: 575–607. 10.1146/annurev.arplant.48.1.575 15012275

[bib23] Herrington M. E., Hardner C., Wegener M., Woolcock L. L., and Dieters M. J., 2011 Rain Damage to Strawberries Grown in Southeast Queensland: Evaluation and Genetic Control. HortScience 46: 832–837. 10.21273/HORTSCI.46.6.832

[bib24] Hirakawa H., Shirasawa K., Kosugi S., Tashiro K., Nakayama S., 2014 Dissection of the Octoploid Strawberry Genome by Deep Sequencing of the Genomes of Fragaria Species. DNA Res. 21: 169–181. 10.1093/dnares/dst049 24282021 PMC3989489

[bib25] Hu Y., Li Y., Hou F., Wan D., Cheng Y., 2018 Ectopic expression of Arabidopsis broad-spectrum resistance gene RPW8.2 improves the resistance to powdery mildew in grapevine (Vitis vinifera). Plant Sci. 267: 20–31. 10.1016/j.plantsci.2017.11.005 29362096

[bib26] Jia Y., Yuan Y., Zhang Y., Yang S., and Zhang X., 2015 Extreme expansion of NBS-encoding genes in Rosaceae. BMC Genet. 16: 48 10.1186/s12863-015-0208-x 25935646 PMC4417205

[bib27] Jupe F., Chen X., Verweij W., Witek K., Jones J. D. G., 2014 Genomic DNA Library Preparation for Resistance Gene Enrichment and Sequencing (RenSeq) in Plants, pp. 291–303 in Plant-Pathogen Interactions: Methods and Protocols, edited by Birch P., Jones J. T., and Bos J. I. B. Humana Press, Totowa, NJ 10.1007/978-1-62703-986-4_22 24643569

[bib28] Jupe F., Pritchard L., Etherington G. J., MacKenzie K., Cock P. J. A., 2012 Identification and localisation of the NB-LRR gene family within the potato genome. BMC Genomics 13: 75 10.1186/1471-2164-13-75 22336098 PMC3297505

[bib29] Khan M., Subramaniam R., and Desveaux D., 2016 Of guards, decoys, baits and traps: pathogen perception in plants by type III effector sensors. Curr. Opin. Microbiol. 29: 49–55. 10.1016/j.mib.2015.10.006 26599514

[bib30] Lai Y., and Eulgem T., 2017 Transcript-level expression control of plant NLR genes. Mol. Plant Pathol. 19: 1267–1281. 10.1111/mpp.12607 28834153 PMC6638128

[bib31] Lukasik E., and Takken F. L. W., 2009 STANDing strong, resistance proteins instigators of plant defence. Curr. Opin. Plant Biol. 12: 427–436. 10.1016/j.pbi.2009.03.001 19394891

[bib32] Lupas A., Van Dyke M., and Stock J., 1991 Predicting Coiled Coils from Protein Sequences. Science 252: 1162–1164. 10.1126/science.252.5009.1162 2031185

[bib33] Mangandi J., Verma S., Osorio L., Peres N.A., van de Weg E., 2017 Pedigree-Based Analysis in a Multiparental Population of Octoploid Strawberry Reveals QTL Alleles Conferring Resistance to *Phytophthora cactorum*. G3: (Bethesda)7: 1707. 10.1534/g3.117.042119 PMC5473751 28592652

[bib34] Metsalu T., and Vilo J., 2015 ClustVis: a web tool for visualizing clustering of multivariate data using Principal Component Analysis and heatmap. Nucleic Acids Res. 43: W566–W570. 10.1093/nar/gkv468 25969447 PMC4489295

[bib35] Miller M. A., Pfeiffer W., and Schwartz T., 2010 Creating the CIPRES Science Gateway for inference of large phylogenetic trees, pp. 1–8 in *Proceedings of the Gateway Computing Environments Workshop (GCE)* IEEE, New Orleans.

[bib36] Munch D., Gupta V., Bachmann A., Busch W., Kelly S., 2018 The Brassicaceae Family Displays Divergent, Shoot-Skewed NLR Resistance Gene Expression. Plant Physiol. 176: 1598–1609. 10.1104/pp.17.01606 29187571 PMC5813569

[bib37] Nellist C. F., Vickerstaff R. J., Sobczyk M. K., Marina-Montes C., Wilson F. M., 2019 Quantitative trait loci controlling Phytophthora cactorum resistance in the cultivated octoploid strawberry (Fragaria × ananassa). Hortic. Res. 6: 60 10.1038/s41438-019-0136-4 31069084 PMC6491645

[bib38] Nepal P. M., Andersen J. E., Neupane S., and Benson V. B., 2017 Comparative Genomics of Non-TNL Disease Resistance Genes from Six Plant Species. Genes (Basel) 8 10.3390/genes8100249 PMC5664099 28973974

[bib39] Peirce J. L., Li H., Wang J., Manly K. F., Hitzemann R. J., 2006 How replicable are mRNA expression QTL? Mamm. Genome 17: 643–656. 10.1007/s00335-005-0187-8 16783644

[bib40] Pincot D. D. A., Poorten T. J., Hardigan M. A., Harshman J. M., Acharya C. B., 2018 Genome-Wide Association Mapping Uncovers *Fw1*, a Dominant Gene Conferring Resistance to Fusarium Wilt in Strawberry. G3: (Bethesda)8: 1817. 10.1534/g3.118.200129 PMC5940171 29602808

[bib41] R. Development Core Team, 2014 R: A language and environment for statistical computing.

[bib42] Rajaraman J., Douchkov D., Hensel G., Stefanato F. L., Gordon A., 2016 An LRR/Malectin Receptor-Like Kinase Mediates Resistance to Non-adapted and Adapted Powdery Mildew Fungi in Barley and Wheat. Front. Plant Sci. 7: 1836 10.3389/fpls.2016.01836 28018377 PMC5156707

[bib43] Roach J. A., Verma S., Peres N. A., Jamieson A. R., van de Weg W. E., 2016 FaRXf1: a locus conferring resistance to angular leaf spot caused by Xanthomonas fragariae in octoploid strawberry. Theor. Appl. Genet. 129: 1191–1201. 10.1007/s00122-016-2695-1 26910360

[bib44] Salinas N., Verma S., Peres N., and Whitaker V.M., 2018 FaRCa1: a major subgenome-specific locus conferring resistance to Colletotrichum acutatum in strawberry. *Theoretical and Applied Genetics*. 10.1007/s00122-018-3263-7 PMC6449309 30564908

[bib45] Stamatakis A., 2014 RAxML version 8: a tool for phylogenetic analysis and post-analysis of large phylogenies. Bioinformatics 30: 1312–1313. 10.1093/bioinformatics/btu033 24451623 PMC3998144

[bib46] Suyama M., Torrents D., and Bork P., 2006 PAL2NAL: robust conversion of protein sequence alignments into the corresponding codon alignments. Nucleic acids research 34 (suppl_2): W609–W612. 10.1093/nar/gkl315 PMC1538804 16845082

[bib47] Sánchez-Sevilla J. F., Vallarino J. G., Osorio S., Bombarely A., Posé D., 2017 Gene expression atlas of fruit ripening and transcriptome assembly from RNA-seq data in octoploid strawberry (Fragaria × ananassa). Sci. Rep. 7: 13737 10.1038/s41598-017-14239-6 29062051 PMC5653846

[bib63] Tang H., Krishnakumar V., and Li J., 2015 jcvi: JCVI utility libraries.

[bib48] Tang Y., Liu X., Wang J., Li M., Wang Q., 2016 GAPIT Version 2: An Enhanced Integrated Tool for Genomic Association and Prediction. Plant Genome 9: 2 10.3835/plantgenome2015.11.0120 27898829

[bib49] Tennessen J. A., Govindarajulu R., Ashman T.-L., and Liston A., 2014 Evolutionary Origins and Dynamics of Octoploid Strawberry Subgenomes Revealed by Dense Targeted Capture Linkage Maps. Genome Biol. Evol. 6: 3295–3313. 10.1093/gbe/evu261 25477420 PMC4986458

[bib50] van Dijk T., Pagliarani G., Pikunova A., Noordijk Y., Yilmaz-Temel H., 2014 Genomic rearrangements and signatures of breeding in the allo-octoploid strawberry as revealed through an allele dose based SSR linkage map. BMC Plant Biol. 14: 55 10.1186/1471-2229-14-55 24581289 PMC3944823

[bib51] Van Ghelder C., and Esmenjaud D., 2016 TNL genes in peach: insights into the post-LRR domain. BMC Genomics 17: 317 10.1186/s12864-016-2635-0 27129402 PMC4851768

[bib52] Verma S., Bassil N. V., van de Weg E., Harrison R. J., Monfort A., 2017 Development and evaluation of the Axiom IStraw35 384HT array for the allo-octoploid cultivated strawberry Fragaria ×ananassa. Acta Hortic. 1156: 75–82.

[bib53] Verma S., Osorio L. F., Lee S., Bassil N. V., and Whitaker V. M., 2018 Genome-Assisted Breeding in the Octoploid Strawberry, pp. 161–184 in The Genomes of Rosaceous Berries and Their Wild Relatives, edited by Hytönen T., Graham J., and Harrison R. Springer International Publishing, Cham 10.1007/978-3-319-76020-9_12

[bib54] Vining K. J., Salinas N., Tennessen J. A., Zurn J. D., Sargent D. J., 2017 Genotyping-by-sequencing enables linkage mapping in three octoploid cultivated strawberry families. PeerJ 5: e3731 10.7717/peerj.3731 28875078 PMC5581533

[bib55] Wen Z., Yao L., Wan R., Li Z., Liu C., 2015 Ectopic Expression in Arabidopsis thaliana of an NB-ARC Encoding Putative Disease Resistance Gene from Wild Chinese Vitis pseudoreticulata Enhances Resistance to Phytopathogenic Fungi and Bacteria. Front. Plant Sci. 6: 1087 10.3389/fpls.2015.01087 26697041 PMC4674559

[bib56] Witek K., Jupe F., Witek A. I., Baker D., Clark M. D., 2016 Accelerated cloning of a potato late blight–resistance gene using RenSeq and SMRT sequencing. Nat. Biotechnol. 34: 656–660. 10.1038/nbt.3540 27111721

[bib57] Xiao S., Ellwood S., Calis O., Patrick E., Li T., 2001 Broad-Spectrum Mildew Resistance in Arabidopsis thaliana Mediated by RPW8. 10.1126/science.291.5501.118 11141561

[bib58] Xiong J.-S., Zhu H.-Y., Bai Y.-B., Liu H., and Cheng Z.-M., 2018 RNA sequencing-based transcriptome analysis of mature strawberry fruit infected by necrotrophic fungal pathogen Botrytis cinerea. Physiol. Mol. Plant Pathol. 104: 77–85. 10.1016/j.pmpp.2018.08.005

[bib59] Yang Z., 2007 PAML 4: phylogenetic analysis by maximum likelihood. Mol. Biol. Evol. 24: 1586–1591. 10.1093/molbev/msm088 17483113

[bib60] Zhong Y., and Cheng Z.-M. M., 2016 A unique RPW8-encoding class of genes that originated in early land plants and evolved through domain fission, fusion, and duplication. Sci. Rep. 6: 32923 10.1038/srep32923 27678195 PMC5039405

[bib61] Zhong Y., Zhang X., and Cheng Z.-M., 2018 Lineage-specific duplications of NBS-LRR genes occurring before the divergence of six Fragaria species. BMC Genomics 19: 128 10.1186/s12864-018-4521-4 29422035 PMC5806312

[bib62] Zurn J. D., Ivors K. L., Whitaker V. M., Knapp S. J., Hummer K. E., 2018 Searching for resistance to soilborne pathogens in cultivated strawberries and the Fragaria supercore. *American Phytopathological Society Annual Meeting*.

